# Supplementary material for: Fine-Tuning Mybl2 Is Required for Proper Mesenchymal-to-Epithelial Transition during Somatic Reprogramming
Source: Cell Rep. 2018 Aug 7;24(6):1496–1511.e8. doi: 10.1016/j.celrep.2018.07.026 (PMC6092268; doi:10.1016/j.celrep.2018.07.026)
Supplement: Document S1. Figures S1–S8 [file mmc1.pdf]

Cell Reports, Volume *24*

Supplemental Information

Fine-Tuning Mybl2 Is Required for Proper Mesenchymal-to-Epithelial Transition during Somatic Reprogramming

Carl Ward, Giacomo Volpe, Pierre Cauchy, Anetta Ptasinska, Ruba Almaghrabi, Daniel Blakemore, Monica Nafria, Doris Kestner, Jon Frampton, George Murphy, Yosef Buganim, Keisuke Kaji, and Paloma García

Figure S1

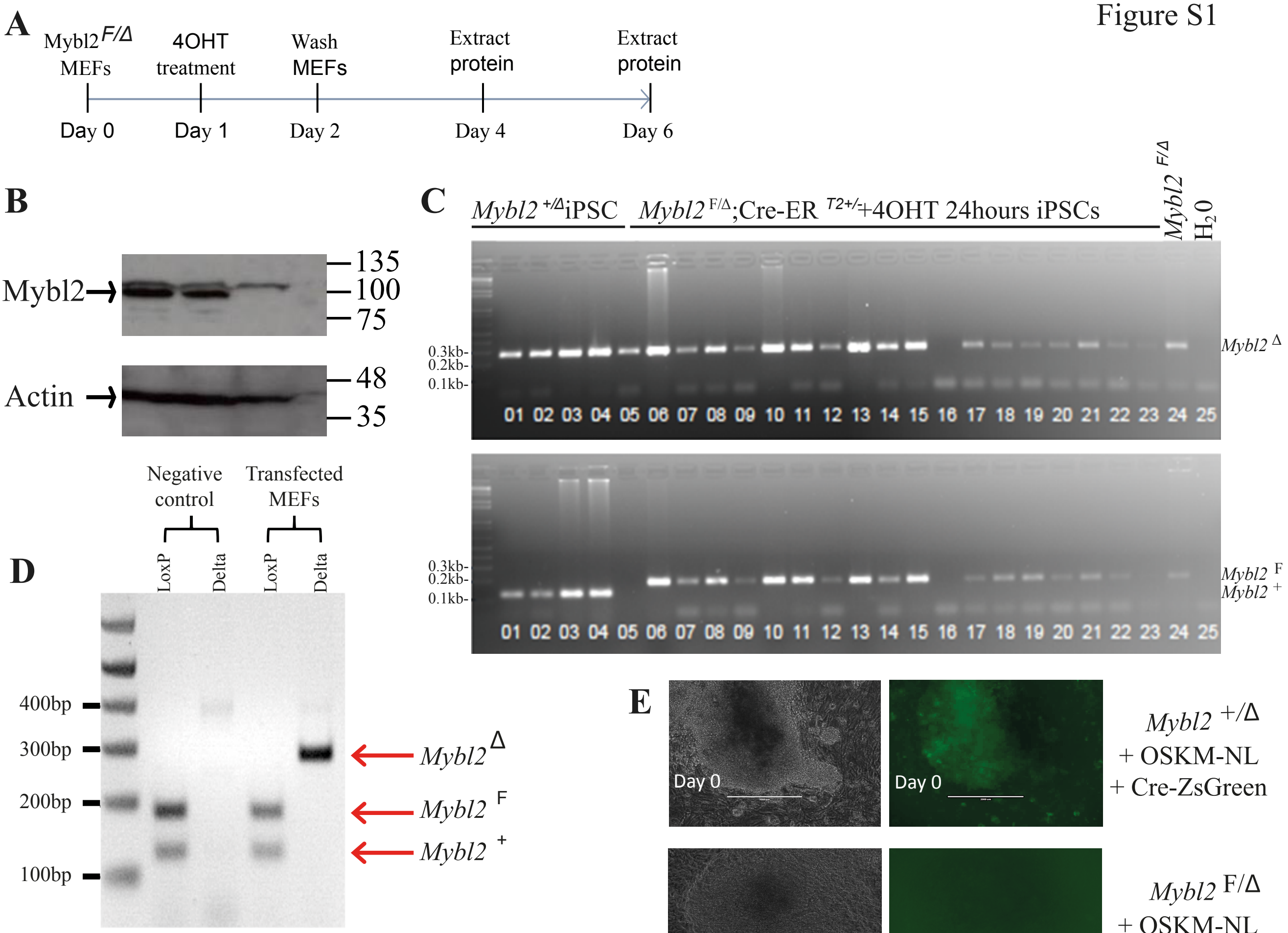

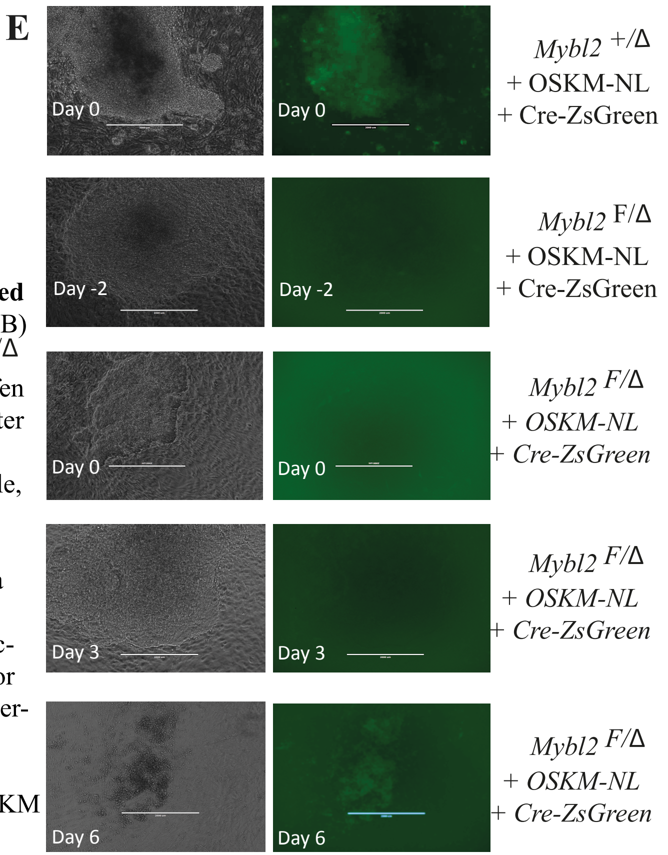

Figure S1. Mybl2 is required for reprogramming, Related to Figure 1. (A) Schematic representation of the strategy. (B) *Mybl2*$^{F/\Delta}$ Cre-ERT2$^{+/-}$ MEFs untreated (Lane 1), *Mybl2*$^{F/\Delta}$ Cre-ERT2$^{+/-}$ MEFs treated with 500nM 4-hydroxytamoxifen (4OHT) for 24h. 48 hours (Lane 2) or 96 hours (Lane 3) after 4OHT treatment. Western blotting with antibodies against Mybl2 or Actin. (C) PCR to detect the deleted *Mybl2*$^{\Delta}$ allele, one fragment at 240bp shows that there is a *Mybl2*$^{\Delta}$ allele present (upper) and PCR to detect the *Mybl2*^{WT} allele and *Mybl2*^{F} alleles, one fragment at 143bp shows that there is a *Mybl2*^{F} allele present (lower panel). (D) Validation of Fu-TetO-CreZsGreen construct. *Mybl2*$^{F/+}$ MEFs were transfected with the Fu-TetO-CreZsGreen or control vector. PCR for detection of the *Mybl2*$^{\Delta}$, *Mybl2*^{F} and *Mybl2*$^{+}$ alleles was performed. (E) Representative images of iPSC colonies from *Mybl2*$^{F/\Delta}$ and *Mybl2*$^{+/\Delta}$ transduced with dox inducible CreZsGreen lentiviral particles (MOI 10) together with OSKM lacking loxP sites (OSKM-NL) (MOI 2).

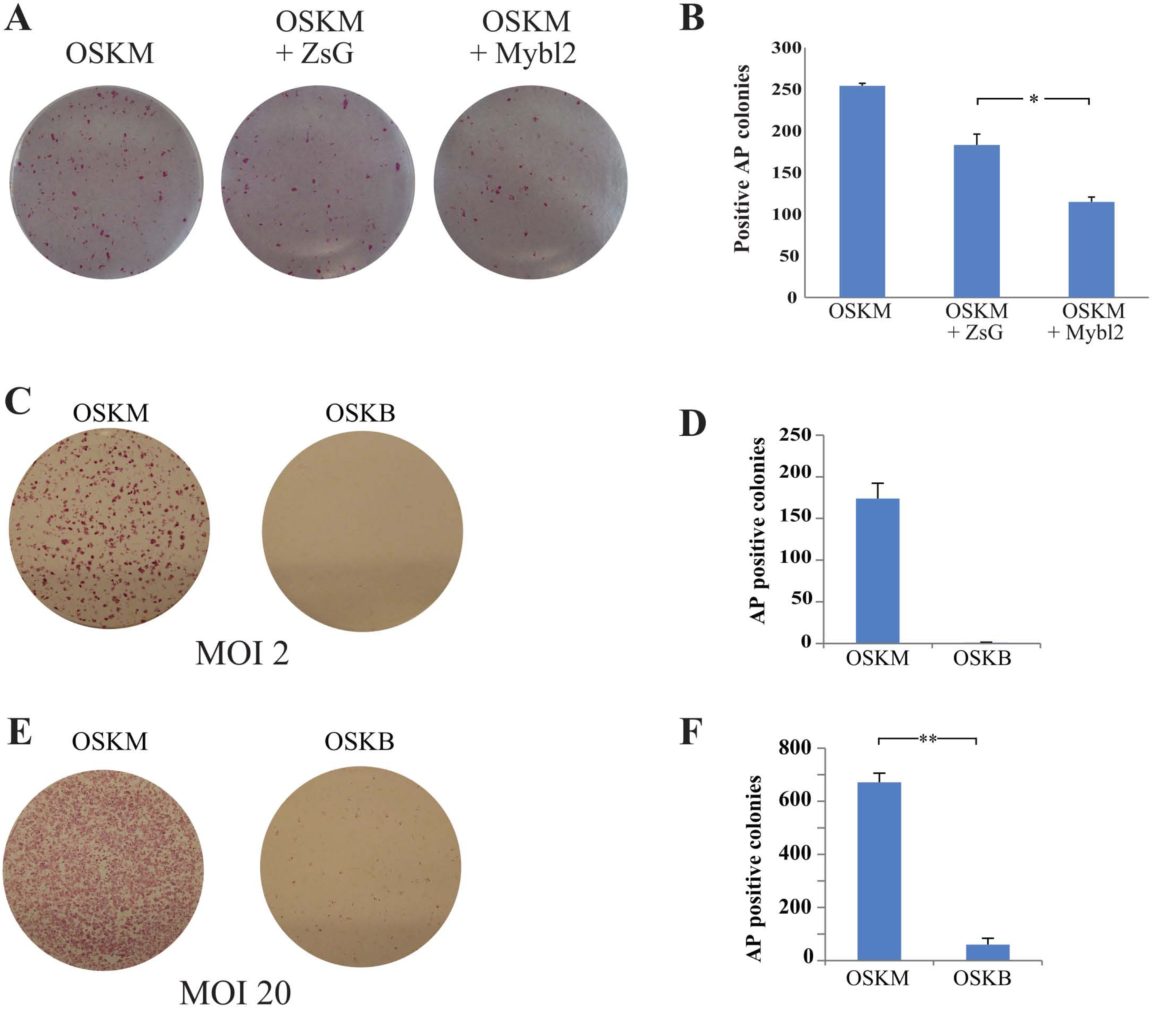

Figure S2. Alkaline phosphatase staining of reprogrammed wt and *p53* $^{-/-}$ MEFs, Related to Figure 2.
(A) MEFs were transduced with OSKM (left panel), OSKM + Zs-Green (middle panel) or OSKM + Mybl2 (right panel) lentiviralparticles (all MOI 1), then tested for pluripotency by alkaline phosphatase staining at day 10. Plates showing positive AP staining (red). (B) Graph representing the positive colony counts. (C) *p53* $^{-/-}$MEFs were transduced with OSKM, OSK or OSKB lentiviral particles at MOI 2 (C-D) or MOI 20 (E-F) and allowed to reprogram for 14 days before staining for AP activity. Quantification of alkaline positive colonies at MOI 2 (D) or MOI 20 (F) are shown on the histograms. N=3 biological replicates. Error bars represent SEM. *<0.05 by unpaired two-tailed T-test.

Figure S3

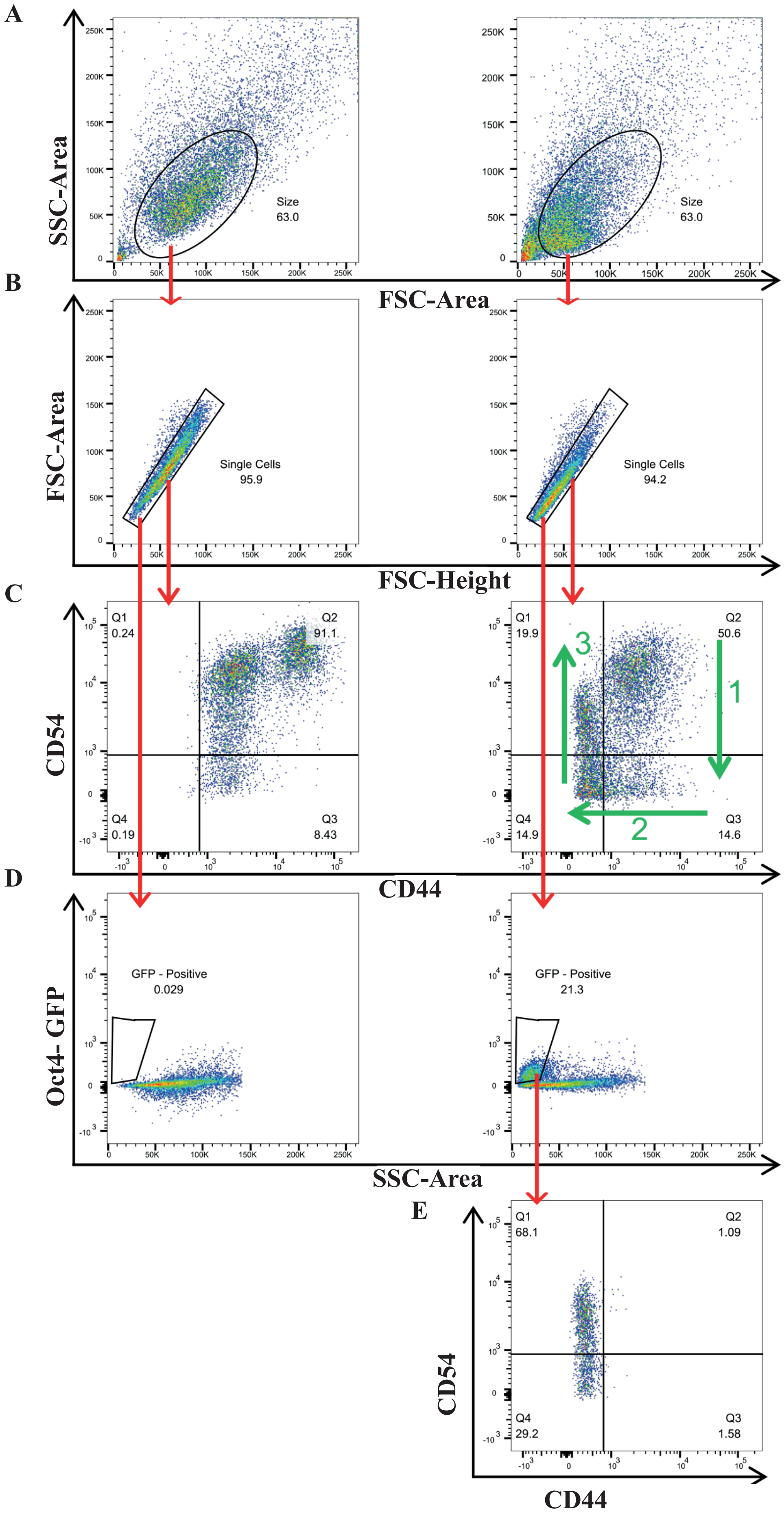

Figure S3. Gating strategy used in flow cytometry for reprogramming kinetics studies, Related to Figure 3.
Cells were gated based on their size and granularity (A, Live), then for doublet discrimination (B), then shown as dot plots representing their expression of CD44 and CD54. Green arrows show progression od CD44/CD54 expression during reprogramming (C). Reprogramming MEFs were gated for GFP expression driven by endogenous Oct4 promoter (D, GFP positive population). (E) Dot plot showing CD44/CD54 expression in GFP positive pre-iPSCs and iPSCs.

Figure S4

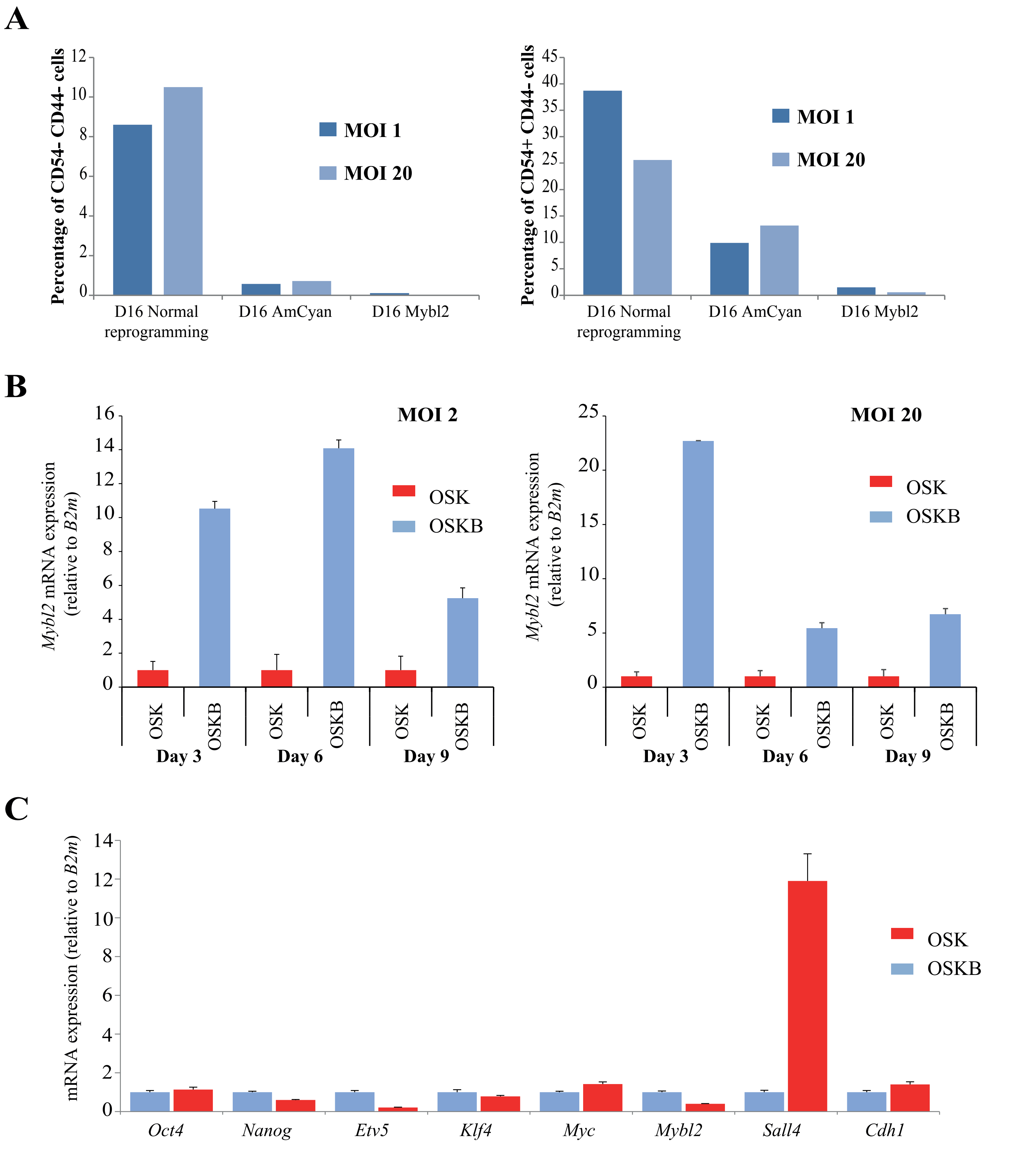

Figure S4. Somatic reprogramming is impaired when Mybl2 is overexpressed, Related to Figure 3.
(A) Somatic reprogramming is impaired using different Mybl2 expression levels but same OSK levels. MEFs were infected with OSK (harbouring Cherry reporter gene) at MOI 1 and Mybl2-AmCyan or AmCyan control lentiviral particles at MOI 1 and MOI 20. 16 days later, CD44/CD54 expression was assessed by flow cytometry. Histograms show the percentage of CD44-/CD54- (pre-iPSCs) and CD44-/CD54+ (iPSCs). (B) Real time PCR showing fold change in *Mybl2* expression levels during somatic reprogramming in OSKB in comparison to OSK sample (data from main Figure 3). Results show gene expression realtive to ESCs expression and normalized to the house keeping gene *β2-microglobulin*. (C) RNA expression of pluripotent genes in an individual iPSC clone derived from OSKB MOI 20 after excision of the lentiviral cassette measured using qRT-PCR.Values are relative to an ESC line and normalized against the *β2-microglobulin* gene. N=3 technical replicates.

Figure S5

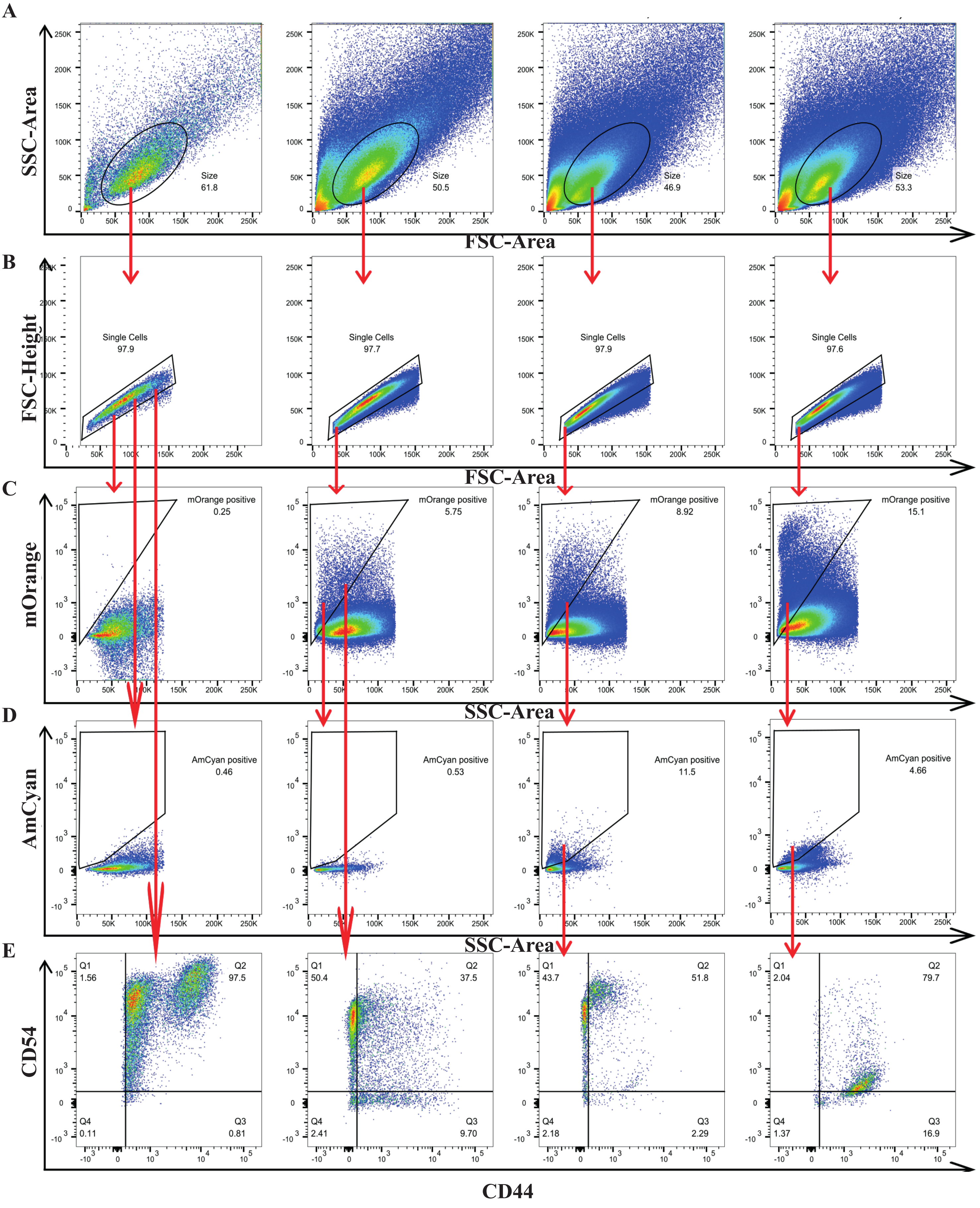

Figure S5. Gating strategy with Tg-reprogrammable MEFs (MKOS), Related to Figure 4.
Cells were gated based on size and granularity to exclude debris (A) and gated to identify single cells (B). Cells were gated for mOrange expression (C), then AmCyan expression (D) and finally shown as a dot plot based on their CD44 and CD54 expression (E).

Figure S6

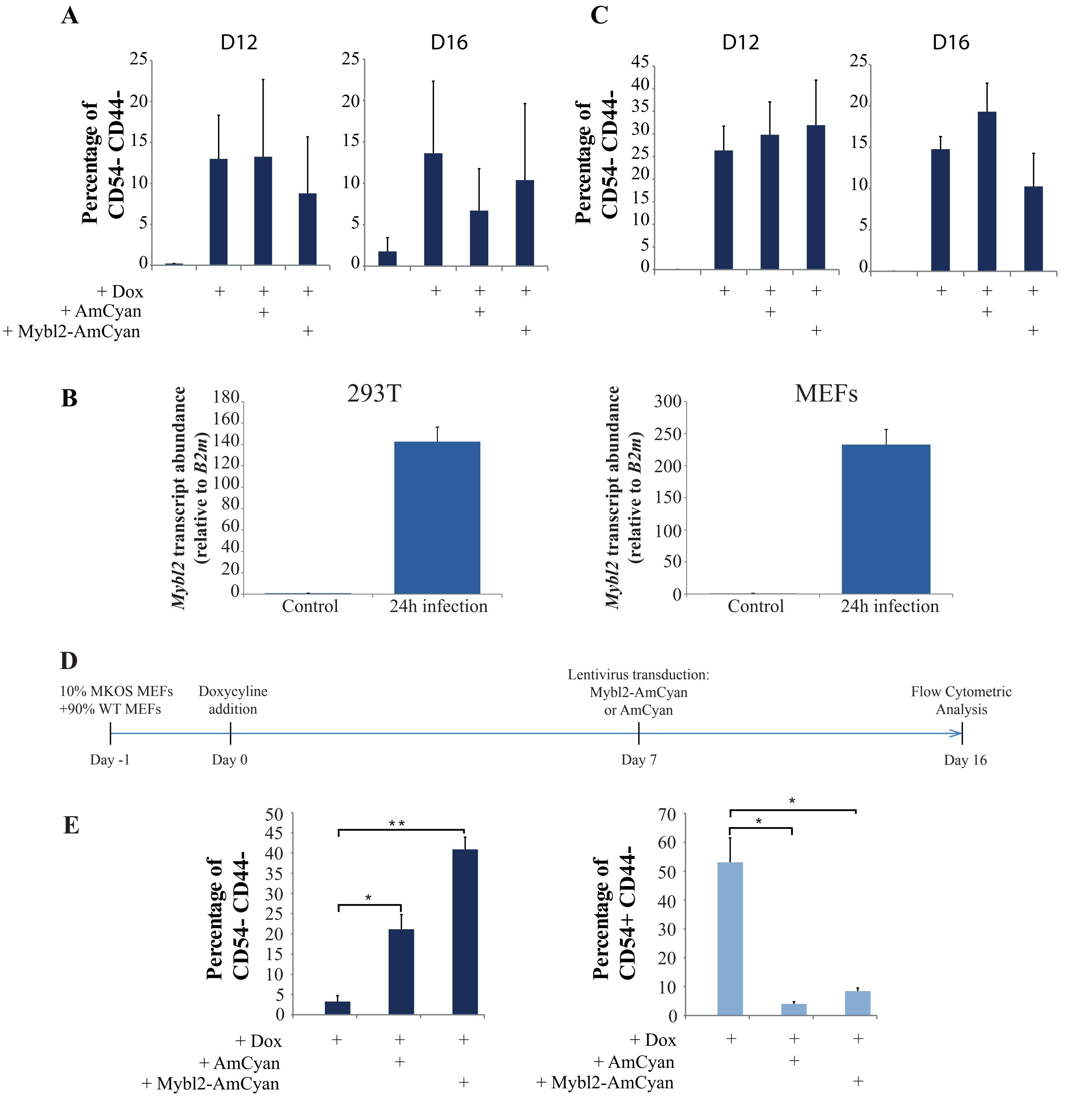

Figure S6. Mybl2 overexpression in Tg reprogrammable MEFs, Related to Figure 4. Graphs represent the percentange of $CD44^-/CD54^-$ cells (pre-iPSCs) at day 12 and 16 of reprogramming for Tg reprogrammable MEFs infected with AmCyan and Mybl2-AmCyan at day -2 before dox addition (A)or infected with AmCyan and Mybl2-Amcyan at day 3 after dox addition (C). N=3 biological replicates. (B) Validation of Mybl2 expression 24 hours after infection in 293T or MEFs (MOI 10). Mybl2 transcript abundance was measured by Taqman-PCR and normalized against the house keeping gene β2-microglobulin. N=1. (D) Schematic representation of the experimental design for Mybl2 infection at day 7 of somatic reprogramming in Tg reprogrammable MEFs. (E) Graphs showing the percentage of $CD44^-/CD54^-$ and $CD44^-/CD54^+$ in $mOrange^+$ (MKOS + dox) or in double positive $mOrange^+$, $AmCyan^+$ population (for MKOS + dox+ AmCyan and MKOS + dox + Mybl2AmCyan) at day 16. N=2 biological replicates.

Figure S7

A

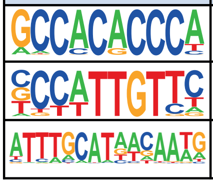

Motif	Match	Score	% targets
	Klf	1e-204	13.75
	Sox	1e-193	29.70
	Oct4-Sox2-Tcf-Nanog	1e-145	10.38

B

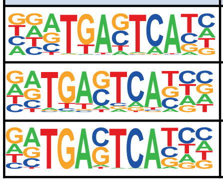

Motif	Match	Score	% targets
	Fra1	1e-537	25.06
	Fosl2	1e-517	18.89
	Jun-AP1	1e-495	15.51

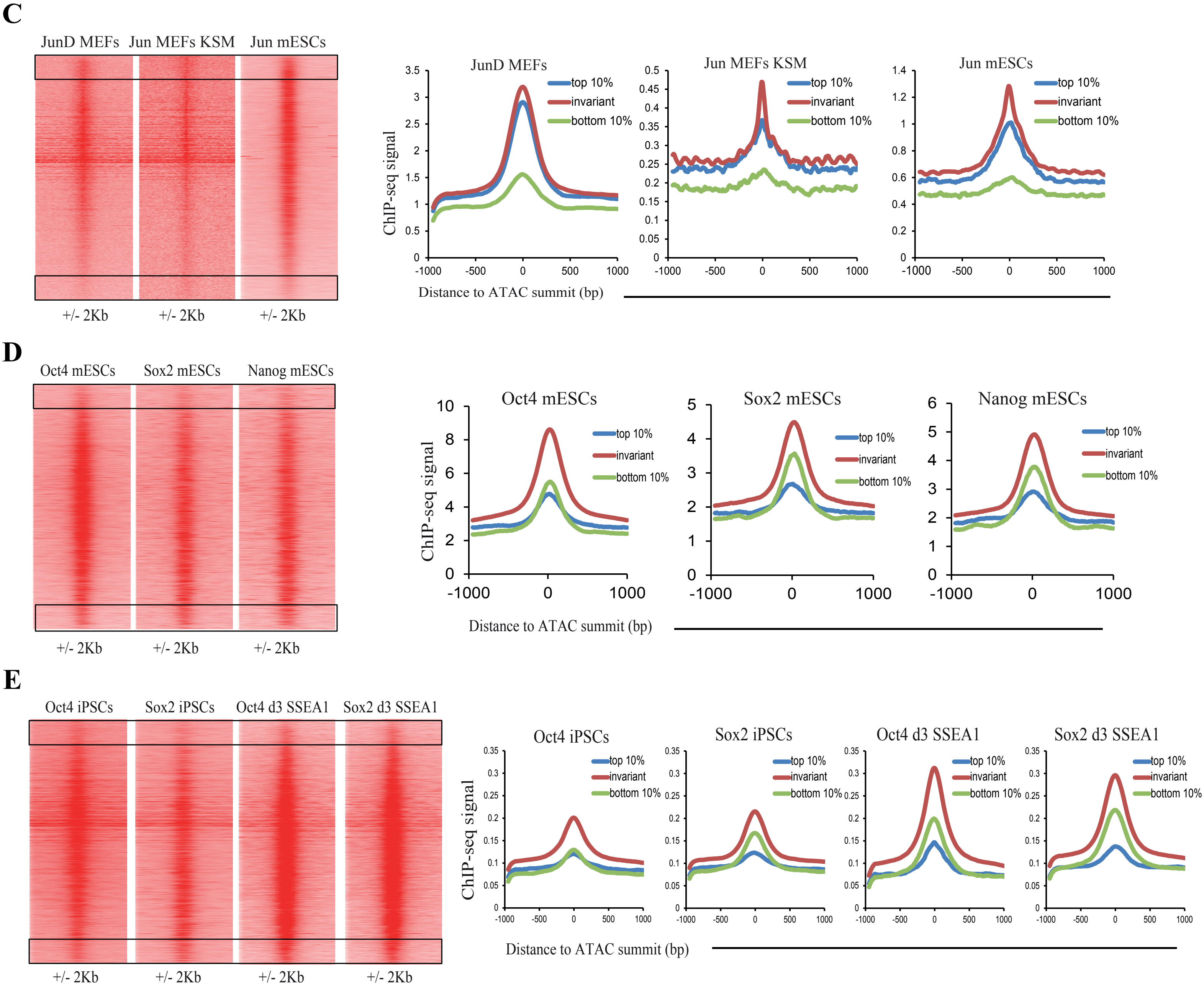

Figure S7. Mybl2 overexpression in Tg reprogrammable MEFs leads to a less accessible chromatin conformation, Related to Figure 5. (A) Motif enrichment in bottom 10% ATAC-seq peaks from union of peaks between control and Mybl2 overexpressing cells at day 3 of reprogramming.(B) Motif enrichment in top 10% ATAC-seq peaks from union of peaks between control and Mybl2 overexpressing cells at day 3 of reprogramming. (C) Heatmaps showing Jun ChIP-seq signal in (mESCs, MEFs, KSM+JunDN and MEFs) by decreasing ATAC-seq tag count signal for Mybl2-treated relative to control (AmCyan). (left) and average Jun ChIP-seq peak signal profiles with respect to the distance to ATAC summit (right). (D) Heatmaps showing Oct-4, Sox2 and Nanog ChIP-seq signal in mESCs, MEFs and iPSCs) by decreasing ATAC-seq tag count signal for Mybl2-treated relative to control (AmCyan) (right) and average Oct-4, Sox2 and Nanog ChIP-seq peak signal profiles with respect to the distance to ATAC summit (left). (E) Heatmaps showing Oct-4 and Sox2 ChIP-seq signal (in MEFs and iPSCs) by decreasing ATAC-seq tag count signal for Mybl2-treated relative to control (AmCyan) (right) and average Oct-4 and Sox2 ChIP-seq peak signal profiles with respect to the distance to ATAC summit (left).

Figure S8

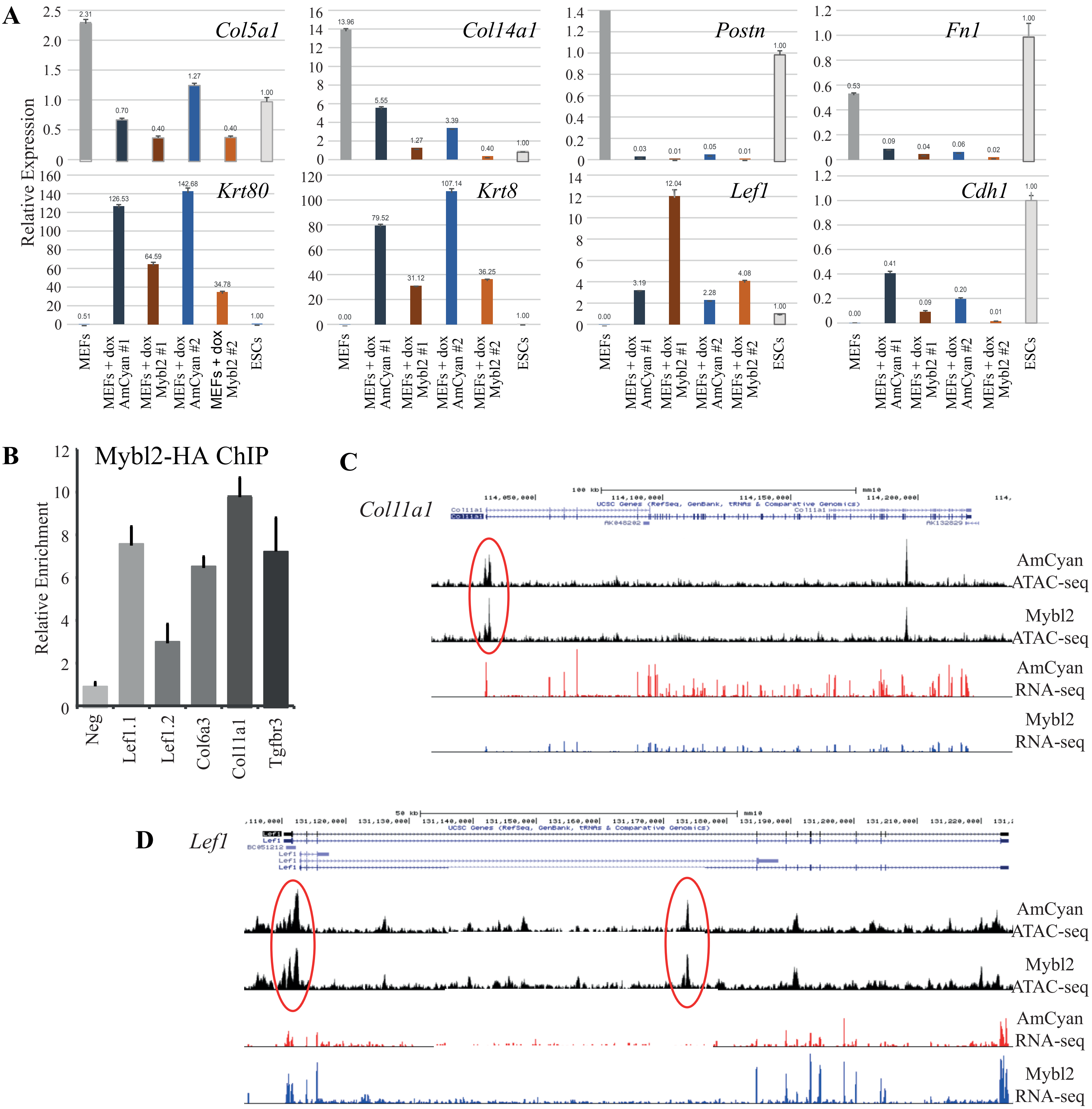

Figure S8. Mybl2 overexpression during reprogramming deregulates mesenchymal-to-epithelial transition (MET), Related to Figure 6. (A) Tg-reprogrammable MEFs were transduced with either AmCyan orMybl2-AmCyan lentivirus and induction of reprogramming was started one day after infection by doxycycline addition. Three days after the starting of reprogramming cells were sorted based on mOrange and AmCyan expression and RNA extracted. Taqman qPCR was done for the selected genes on two different RNA sets from the RNA-seq as well as for MEFs and ESCs. Results show relative gene expression normalized to the house keeping gene *β2-microglobulin.* 3 technical replicates and two biological replicates (set A and set B). ATAC-seq, and RNA-seq UCSC genome browser tracks for *Col11a1*(B) and *Lef1* C). Red circles show differential ATAC-seq peaks between both conditions. D) Representative X-ChIP on immortalized MEFs infected with Mybl2-HA-AmCyan using HA antibody. Enrichment fold was determined against negative control region. Graphs illustrate level of enrichment for each binding site identified for *Tgfbr3, Lef1, Col11a1, and Col6a2.*
