# Supplementary material for: Fine-Tuning Mybl2 Is Required for Proper Mesenchymal-to-Epithelial Transition during Somatic Reprogramming
Source: Cell Rep. 2018 Aug 7;24(6):1496–1511.e8. doi: 10.1016/j.celrep.2018.07.026 (PMC6092268; doi:10.1016/j.celrep.2018.07.026)
Supplement: Document S2. Article plus Supplemental Information [file mmc2.pdf]

Cell Reports

Article

Fine-Tuning Mybl2 Is Required for Proper Mesenchymal-to-Epithelial Transition during Somatic Reprogramming

Graphical Abstract

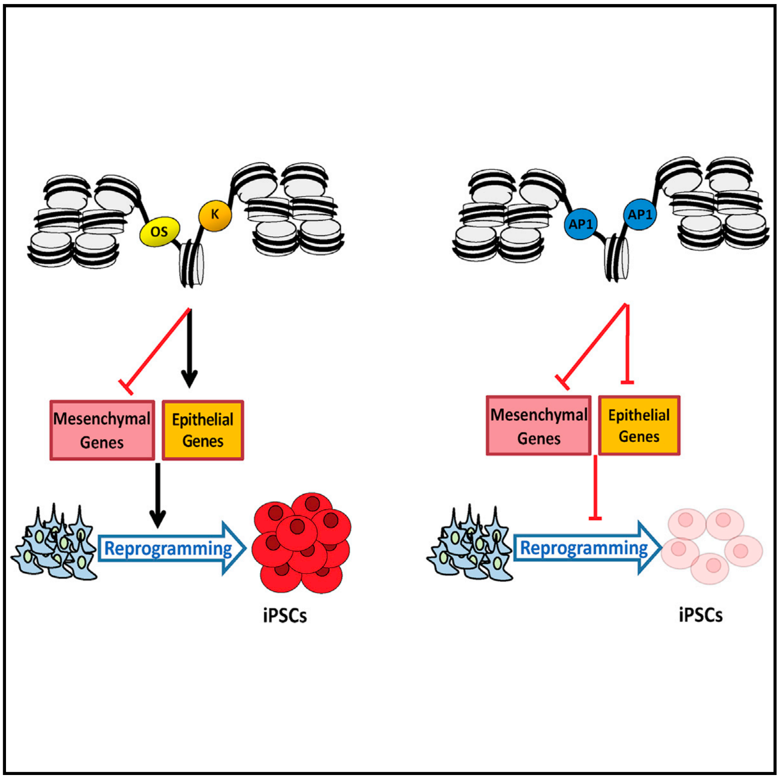

Highlights

- Deletion and overexpression of MYBL2 pluripotency factor inhibit somatic reprogramming
- Mybl2 overexpression affects the accessibility of pioneer factors to the chromatin
- Mybl2 overexpression promotes accessibility of reprogramming blockers to the chromatin
- High Mybl2 levels deregulate key genes for proper MET, a requirement for reprogramming

Authors

Carl Ward, Giacomo Volpe, Pierre Cauchy, ..., Yosef Buganim, Keisuke Kaji, Paloma García

Correspondence

p.garcia@bham.ac.uk

In Brief

Ward et al. show that Mybl2 expression level is a gatekeeper for the initiation of reprogramming. They find that Mybl2 overexpression leads to changes in the chromatin landscape, affecting the accessibility of pioneer factors to the chromatin and promoting accessibility for the AP1 family of transcription factors, known to be reprogramming blockers.

Ward et al., 2018, Cell Reports *24*, 1496–1511
August 7, 2018 © 2018 The Authors.
https://doi.org/10.1016/j.celrep.2018.07.026

OPEN ACCESS
CellPress

Cell Reports
Article

Fine-Tuning Mybl2 Is Required for Proper Mesenchymal-to-Epithelial Transition during Somatic Reprogramming

Carl Ward,[1] Giacomo Volpe,[1] Pierre Cauchy,[1,2] Anetta Ptasinska,[1] Ruba Almaghrabi,[1] Daniel Blakemore,[1] Monica Nafria,[1] Doris Kestner,[1] Jon Frampton,[1] George Murphy,[3] Yosef Buganim,[4] Keisuke Kaji,[5] and Paloma García[1,6,*]
[1]Institute of Cancer and Genomic Science, College of Medical and Dental Sciences, University of Birmingham, Birmingham, UK
[2]Department of Molecular and Cellular Immunology, Max Planck Institute of Immunobiology and Epigenetics, Freiburg, Germany
[3]Department of Medicine, Boston University School of Medicine, Boston, MA, USA
[4]The Institute for Medical Research Israel-Canada, The Hebrew University Hadassah Medical School, Jerusalem, Israel
[5]MRC Centre for Regenerative Medicine, University of Edinburgh, Edinburgh, UK
[6]Lead Contact
*Correspondence: p.garcia@bham.ac.uk
https://doi.org/10.1016/j.celrep.2018.07.026

SUMMARY

During somatic reprogramming, Yamanaka's pioneer factors regulate a complex sequence of molecular events leading to the activation of a network of pluripotency factors, ultimately resulting in the acquisition and maintenance of a pluripotent state. Here, we show that, contrary to the pluripotency factors studied so far, overexpression of Mybl2 inhibits somatic reprogramming. Our results demonstrate that Mybl2 levels are crucial to the dynamics of the reprogramming process. Mybl2 overexpression changes chromatin conformation, affecting the accessibility of pioneer factors to the chromatin and promoting accessibility for early immediate response genes known to be reprogramming blockers. These changes in the chromatin landscape ultimately lead to a deregulation of key genes that are important for the mesenchymal-to-epithelial transition. This work defines Mybl2 level as a gatekeeper for the initiation of reprogramming, providing further insights into the tight regulation and required coordination of molecular events that are necessary for changes in cell fate identity during the reprogramming process.

INTRODUCTION

Somatic reprogramming can be achieved through overexpression of the exogenous factors Oct4, Sox2, Klf4, and Myc (OSKM), first described by Takahashi and Yamanaka (Takahashi and Yamanaka, 2006). Since then, several reports have provided detailed descriptions of the molecular dynamics of the reprogramming process (Buganim et al., 2012; Hansson et al., 2012; Li et al., 2010; O'Malley et al., 2013; Polo et al., 2012). Reprogramming occurs in an organized way, with rapid genome-wide transcriptional changes during the first days induced by OSKM overexpression.

Despite the recent increase in knowledge about factors that enhance reprogramming such as Nanog (Theunissen et al., 2011), Lin28 (Viswanathan et al., 2008), and chromatin remodelers (Onder et al., 2012), and specific barriers that contribute to lower reprogramming efficiency such as H3K9 methylation (Chen et al., 2013) and upregulation of p53 and p21 (Banito and Gil, 2010; Marión et al., 2009), the specific contribution of each endogenous pluripotency factor to the reprogramming process remains unknown. Elucidating the individual contributions of pluripotency factors and epigenetic remodelers to the reprogramming process is crucial to improve reprogramming efficiency and to produce clinical grade induced pluripotent stem cells (iPSCs) for their use in regenerative medicine.

Among the genes that are upregulated during reprogramming is that encoding the transcription factor Mybl2. Despite its general role in the regulation of proliferation (Musa et al., 2017) and genome stability throughout the animal kingdom (Ahlbory et al., 2005; Fung et al., 2002; García and Frampton, 2006; Manak et al., 2002; Shepard et al., 2005; Tarasov et al., 2008), *Mybl2* has been proposed to have a function as a pluripotency gene. *Mybl2* expression is between 1,000- and 10,000-fold higher in embryonic stem cells (ESCs), iPSCs, and trophoectoderm stem cells than in somatic cells (Tarasov et al., 2008; Benchetrit et al., 2015) and is downregulated during embryogenesis (Sitzmann et al., 1996). Similar to *Oct4* or *Sox2* knockout phenotypes, mice lacking *Mybl2* fail to develop past the blastocyst stage as a result of impaired proliferation of the inner cell mass (Tanaka et al., 1999). Moreover, it has been recently demonstrated that Mybl2 is a chromatin-bound partner for Oct4, Sox2, and Nanog in ESCs (Rafiee et al., 2016). Taken together, this compelling evidence suggests that Mybl2 forms part of a pluripotency network. In agreement with this, *Mybl2* RNA levels increase gradually from day 5 of reprogramming, which is even earlier than those encoding other pluripotency factors such as *Nanog* (O'Malley et al., 2013; Polo et al., 2012). How Mybl2 levels affect the reprogramming process and its exact role during reprogramming remain unknown, however.

Here, we demonstrate that correct Mybl2 levels are critical for reprogramming because both its deletion and overexpression

 © 2018 The Authors.
This is an open access article under the CC BY license (http://creativecommons.org/licenses/by/4.0/).

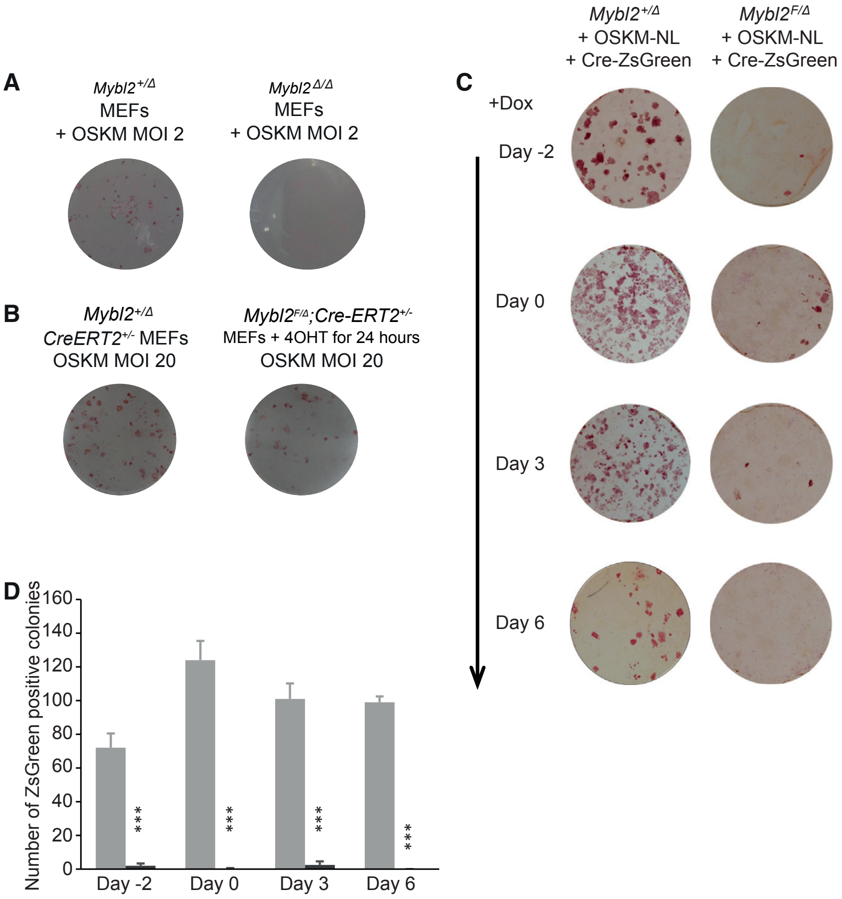

Figure 1. Mybl2 Deletion Inhibits Somatic Reprogramming

(A) *Mybl2*$^{F/\Delta}$ or *Mybl2*$^{+/\Delta}$ were nucleofected with a plasmid that encoded *Cre*-IRES-*EGFP*. GFP^{+} MEFs were sorted, replated, and then transduced with OSKM lentivirus. After 14 days, the MEFs were stained for alkaline phosphatase activity.

(B) *Mybl2*$^{F/\Delta}$*:Cre-ERT2*$^{+/-}$ and *Mybl2*$^{F/\Delta}$*:Cre-ERT2*$^{+/-}$ MEFs were treated with 500 nm 4-OHT for 24 hr and then cultured for a further 96 hr before being transduced with OSKM lentiviral particles at MOI 2 or MOI 20. The MEFs were cultured for 12 days before being stained for alkaline phosphatase activity. Colonies with alkaline phosphatase activity are displayed in red. *Mybl2*$^{F/\Delta}$ or *Mybl2*$^{+/\Delta}$ were transduced with dox-inducible Cre-ZsGreen lentiviral particles (MOI 10) together with OSKM lacking loxP sites (OSKM-NL) (MOI 2) to avoid its excision after dox addition. In the case of the day −2 condition, the cells were transduced with Cre-ZsGreen virus 2 days before infection with OSKM-NL. Dox was added at different times during the reprogramming process (days −1, 0, 3, and 6) and then tested for pluripotency by scoring ZsGreen^{+} colonies under the microscope and by alkaline phosphatase staining at day 10.

(C) Plates showing positive AP staining (red) at day 10.

(D) Graph representing the ZsGreen^{+} colony counts. n = 3 biological replicates. Error bars represent SEMs. ***$p < 0.001$ by unpaired two-tailed t test.

See also Figure S1.

impair the reprogramming process. It is surprising that exogenous expression of the reprogramming factors (OSK) is not sufficient to upregulate the pluripotency program in the presence of high levels of Mybl2. Overexpression of Mybl2 during the early stages of the reprogramming process disturbs the chromatin landscape and the accessibility of OSK to specific chromatin regions affecting the dynamics of the molecular events required for reprogramming to occur.

RESULTS

Mybl2$^{\Delta/\Delta}$ MEFs Are Unable to Reprogram

Given the role of Mybl2 in proliferation and senescence, we sought to study whether reprogramming would be possible in its absence. For this purpose, *Mybl2* floxed allele was deleted using a Cre-recombinase-GFP system in *Mybl2*$^{F/\Delta}$, using *Mybl2*$^{+/\Delta}$ mouse embryonic fibroblasts (MEFs) as a control (García et al., 2005). The GFP^{+} MEFs were subsequently transduced with STEMCCA (Sommer et al., 2009, 2010) (OSKM) lentiviral particles to initiate reprogramming, and after 14 days the cells were stained for alkaline phosphatase (AP) activity. Fewer AP^{+} colonies were detected in the *Mybl2*$^{\Delta/\Delta}$ MEFs compared to *Mybl2*$^{+/\Delta}$ MEFs. To avoid stressing the MEFs by transfection and cell sorting, this experiment was repeated using MEFs from an *Mybl2*$^{F/\Delta}$:*CreERT2* strain in which nuclear translocation of Cre-recombinase was induced by 4-hydroxy tamoxifen (4-OHT) treatment (Figure 1B). Loss of Mybl2 protein was confirmed 96 hr after 4-OHT treatment. *Mybl2*$^{+/\Delta}$*:Cre-ER*$^{T2+/-}$ or *Mybl2*$^{F/\Delta}$*:Cre-ERT2*$^{+/-}$ MEFs were treated for 24 hr with 4-OHT and cultured for 96 hr before replating and transducing with OSKM lentiviral particles. After 12 days, the cells were stained for AP activity. A reduction in the total number of AP^{+} colonies was observed in the 4-OHT-treated *Mybl2*$^{F/\Delta}$*:Cre-ER*$^{T2+/-}$ compared to the *Mybl2*$^{+/\Delta}$*:Cre-ERT2*$^{+/-}$ MEFs (43 ± 23 versus 82 ± 16 colonies, respectively). A possible explanation for the presence of AP colonies in the 4-OHT-treated *Mybl2*$^{F/\Delta}$*:Cre-ERT2*$^{+/-}$ plate could be the inefficient deletion of the floxed (F) allele; thus, all individual iPSC colonies obtained from two different experiments (a total of 50 colonies) were isolated and genotyped, confirming the presence of the *Mybl2*^{F} allele. The absence of the *Mybl2*$^{\Delta/\Delta}$ colonies isolated from different experiments suggests that Mybl2 expression is required for reprogramming to occur. We also determined whether lack of Mybl2 expression was detrimental at a specific time window of the reprogramming process. We infected *Mybl2*$^{F/\Delta}$ and *Mybl2*$^{+/\Delta}$ MEFs with STEMCCA lentivirus lacking loxP sites (OSKM-NL) together with a doxycycline (dox)-inducible Cre-ZsGreen lentivirus (1 day before dox addition). The addition of dox at different times would activate Cre expression, leading to the deletion of the *Mybl2*^{F} allele (Figure S1D). We also infected cells with OSKM-NL 2 days after dox addition (day −2) to delete the *Mybl2*^{F} allele before the initiation of reprogramming. We observed that dox addition at different days before, at the

same time, or after OSKM-NL infection (days −2, 0, 3, and 6, respectively) was detrimental to the reprogramming process (Figures 1C and 1D). Moreover, the few colonies observed on the *Mybl2*$^{F/\Delta}$ plates showed negative staining for ZsGreen, thus indicating that these colonies came from MEFs in which the deletion of the *Mybl2*^{F} allele did not occur (Figure S1E).

Mybl2 Overexpression Impairs Reprogramming

Given the block in reprogramming observed in the absence of Mybl2 expression, we next sought to determine whether Mybl2 overexpression would have a positive impact on reprogramming efficiency, as described for other known pluripotency factors such as Nanog (Theunissen et al., 2011). In this endeavor, MEFs were transduced with OSKM + ZsGreen or OSKM + Mybl2 lentiviral particles, and AP activity was assayed after 10 days. We unexpectedly found that there were visibly fewer AP^{+} colonies when the Mybl2 lentiviral particles were included (OSKM + Mybl2 115 ± 10 compared to 183 ± 23 colonies when reprogramming with OSKM + ZsGreen viruses) (Figures S2A and S2B).

To confirm that Mybl2 overexpression impairs reprogramming, we designed a polycistronic lentiviral vector containing *Oct4*, *Sox2*, *Klf4*, and *Mybl2* (OSKB). Oct4-GFP MEFs were transduced with OSKM, OSK, or OSKB lentiviruses at MOI 2, and ESC-like colony formation, GFP signal, and AP activity were assayed at day 14 (Figure 2A). As expected, considering the importance of Myc in reprogramming efficiency, the number of AP^{+} colonies was visibly lower in OSK-transduced MEFs compared to OSKM-transduced MEFs (Figures 2B and 2C). We were surprised that many fewer colonies were observed in MEFs transduced with OSKB (Figures 2B–2D). We also took advantage of the Oct4-GFP knockin MEFs and determined the expression of GFP in the iPSC colonies generated (an indication of cells that have switched on the endogenous Oct4 promoter) (Lengner et al., 2007). The number of GFP^{+} colonies was highest when reprogramming with OSKM (130 ± 29) compared to OSK (54 ± 18) and OSKB (1 ± 0; Figures 2D and 2E). These striking results suggest that Mybl2 overexpression is detrimental to the overall reprogramming process.

With the aim of forcing the reprogramming process, the number of viral particles was increased by 10-fold (MOI 20). An increase in the number of colonies was observed with each of the different lentiviruses used, but still the number of colonies obtained when reprogramming was performed with OSKB lentivirus was much lower (Figure 2C). Similar to MOI 2, we observed that OSKM and OSK were able to reprogram much more efficiently and with a higher number of GFP^{+} colonies than OSKB (OSKM = 333 ± 124, OSK = 347 ± 118, OSKB = 62 ± 23; Figure 2D). When scoring for GFP^{+} colonies, there was a higher number of GFP positive colonies when reprogramming with OSKM (142 ± 45) compared to OSK (135 ± 39) and OSKB (46 ± 20; Figure 2D).

Given that Oct4 is expressed at an early stage of reprogramming, it could be possible that the colonies scored as GFP^{+} would be not fully mature iPSCs (Buganim et al., 2012). Thus, we sought to confirm our results using another reporter transgene, Nanog-GFP MEFs (Chambers et al., 2007). In these settings, we observed a lower reprogramming efficiency in the Nanog-GFP MEFs compared to Oct4-GFP MEFs, with the OSKM being the most efficient at reprogramming, while the OSKB was barely able to reprogram at all (OSKM = 71 ± 5, OSK = 30 ± 17, OSKB = 5 ± 2; Figure 2E). The number of Nanog-GFP^{+} colonies was 38 ± 4, 18 ± 10, and 3 ± 2 when reprogramming with OSKM, OSK, or OSKB, respectively (Figure 2E). By increasing the number of lentiviral particles by 10-fold we were able to enhance the efficiency of reprogramming, but OSKB reprogramming was still inefficient (Figure 2E). The number of GFP^{+} colonies when reprogramming with OSKM was slightly higher with OSKM and OSK compared to OSKB (OSKM = 27 ± 2, OSK = 29 ± 15, OSKB = 14 ± 7; Figure 2E). Of note, the reprogramming cultures were left for more than 26 days to account for a slower timing of OSKB reprogramming, but even 26 days after OSKB transduction there was no increase in the number of colonies obtained (data not shown).

Moreover, we found that impairment of reprogramming when Mybl2 is included in the reprogramming cocktail is not due to MEFs entering cellular senescence, cell-cycle arrest, or apoptosis, since cells were negative for the β-galactosidase (Figure 2F), did not undergo significant changes in the expression of p21, p53, and p16 genes (Figures 2G and 2H), and displayed a similar percentage of apoptotic nuclei by the terminal deoxynucleotidyl transferase deoxyuridine triphosphate nick end labeling (TUNEL) assay (Figures 2I–2K). Furthermore, to determine whether the reduced reprogramming efficiency was mediated by p53, we transduced *p53*$^{-/-}$ MEFs with OSKM or OSKB lentiviral particles at MOI 2 or MOI 20 and measured AP activity at day 14 (Figures S2C–S2F). The mean number of colonies when *p53*$^{-/-}$ MEFs were transduced with OSKB at MOI 2 remained close to zero (Figure S2D). By increasing the number of viral particles by 10-fold (MOI 20), a large increase in the number of AP colonies could be observed when *p53*$^{-/-}$ MEFs were transduced with OSKM (671 ± 35) but not in OSKB-transduced *p53*$^{-/-}$ MEFs (61 ± 23 AP colonies; Figure S2F).

The above results using different lentiviral vector approaches and different pluripotent reporter MEFs suggest that inclusion of Mybl2 in the reprogramming cocktail dramatically reduces the efficiency of reprogramming, and that this is independent of p53 and cannot be attributed to cell-cycle arrest, apoptosis, or senescence.

CD44/CD54 Reprogramming Kinetics Are Blocked in MEFs Transduced with Mybl2 and Yamanaka Factors

Next, we aimed to identify the precise time at which addition of Mybl2 would affect the reprogramming process. For this purpose, MEFs were transduced with OSKM, OSK, or OSKB lentiviral particles and tracked throughout the reprogramming process using flow cytometry to measure the expression of surface antigens CD44 and CD54 (*Icam1*) (O'Malley et al., 2013) (see gating strategy in Figure S3).

MEFs were tracked through reprogramming at days 6, 9, 12, and 16 after being transduced with OSKM, OSK, or OSKB lentiviral particles at MOI 2. At days 6 and 9, only OSKM-MEFs contained CD44^{-}/CD54^{+} cells (Figure 3A) as the OSK and OSKB remained comparable to non-transduced MEFs. The percentage of CD44^{-}/CD54^{+} cells rose further at days 12 and 16 for OSKM-MEFs and OSK MEFs; however, the OSKB-transduced MEFs

OPEN
ACCESS
CellPress

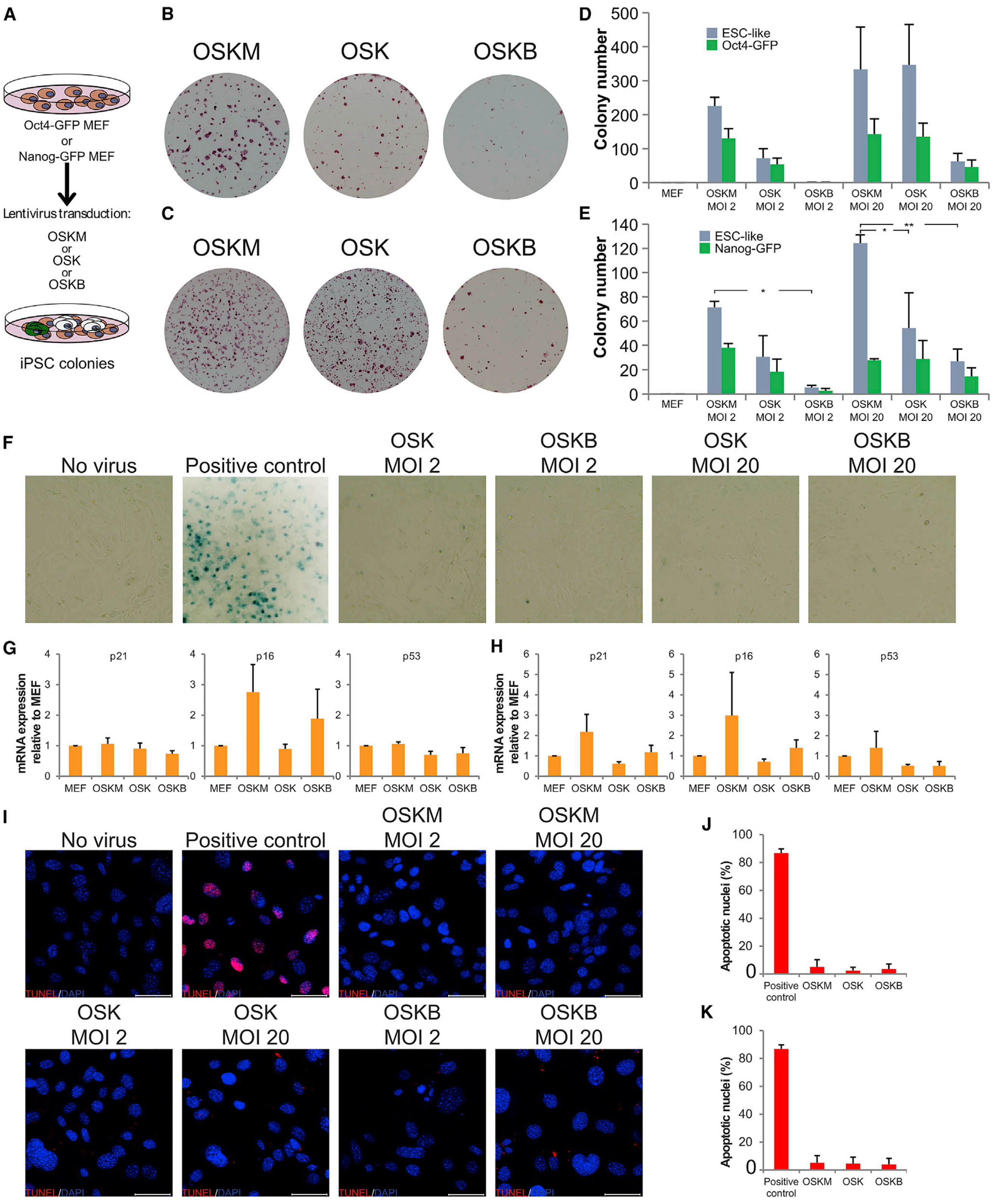

Figure 2. Mybl2 Overexpression Inhibits Somatic Reprogramming

(A) Schematic representation of the experimental procedure. Oct4-GFP or Nanog-GFP MEFs were transduced with MOI 2 or MOI 20 OSKM, OSK, or OSKB lentiviral particles and allowed to reprogram for 14 days before GFP^+ colonies were counted. The cells were stained for alkaline phosphatase activity.

(legend continued on next page)

displayed a significantly lower percentage comparable to non-transduced MEFs (Figure 3A).

Analysis of the reprogramming kinetics in OSKM-, OSK-, or OSKB- (MOI 20) transduced MEFs was performed and demonstrated the presence of $CD44^-/CD54^+$ from day 6 in OSKM-MEFs (Figure 3B), while a small increase in the percentage of $CD44^-/CD54^+$ cells was observed in OSK at day 9 or in OSKB MEFs. By day 12, the proportion of those cells had increased in all three conditions (Figure 3B). Finally, at day 16, we observed reprogramming in all three conditions; however, there was a significantly lower percentage of $CD44^-/CD54^+$ cells in the OSKB- (5.27% ± 0.85%) compared to the OSKM- (13.6% ± 1.1%) or OSK- (14.6% ± 0.89%) transduced MEFs (Figure 3B). These data corroborate the colony number data because it is possible to reprogram with OSKB at MOI 20 but at a reduced efficiency compared to OSKM and OSK reprogramming.

By measuring the expression of CD44 and CD54 at days 6, 9, 12, and 16 of reprogramming, we sought to determine the percentage of partially reprogrammed iPSCs (pre-iPSCs, $CD44^-$, and $CD54^-$), as shown in Figure 3C. Using this approach, we observed 10% of pre-iPSCs at days 9 and 12 in MOI 2 OSKM-MEFs and determined an increase at these time points in OSK-MEFs, while OSKB-MEFs were not able to progress to a pre-iPSC stage and remained at a percentage that is comparable to non-transduced MEFs (Figure 3C).

A similar pattern was observed when the MOI of the reprogramming lentiviruses was increased to 20. Under these conditions, analysis of the reprogramming kinetics revealed a larger percentage of pre-iPSCs by day 6 for OSKM MEFs (Figure 3D). The percentage of pre-iPSCs was lower in OSKB compared to OSKM at all four time points, this percentage being significantly lower at days 6, 9, and 12. To determine whether the increase in reprogramming observed at MOI 20 was caused by the higher expression of OSK factors, we decided to fine-tune the expression of Mybl2, keeping the levels of OSK constant. By infecting the cells with OSK at MOI 1 and Mybl2-AmCyan or AmCyan control at MOI 1 or MOI 20, we could determine the percentage of iPSCs formed that are $Cherry^+$ (infected with OSK) and $AmCyan^+$ (infected with either Mybl2-AmCyan or AmCyan control). Our analysis revealed that infecting Mybl2-AmCyan at either MOI 1 or MOI 20 had a detrimental effect on reprogramming, and by day 16, virtually no $CD44^-/CD54^+$ cells were detected (Figure S4A).

These results demonstrate that an alteration in the reprogramming kinetics occurs when Mybl2 is included as one of the reprogramming factors and suggest that the block in reprogramming occurs at an early stage in the process.

Early and Late Pluripotency Genes Fail to Be Upregulated during Reprogramming in the Presence of Mybl2

We then decided to determine whether the changes observed in the reprogramming efficiency and kinetics in OSKB-transduced MEFs could be the result of Mybl2 affecting the expression of a number of genes involved in pluripotency, cell proliferation, or the mesenchymal-to-epithelial transition (MET). With this purpose, expression of the genes was measured by qRT-PCR at days 3, 6, and 9 during reprogramming with OSK or OSKB lentiviral particles at MOI 2 and MOI 20. We compared OSK to OSKB because of their similar reprogramming kinetics caused by the omission of the *Myc* gene. Expression was calculated as relative to the normal mouse ESC expression for each gene, except *Thy1*, which was set relative to MEF expression because their expression is not detectable in ESCs.

In agreement with our findings on the lack of pre-iPSCs and fully reprogrammed iPSCs, we found that the expression *of Nanog, Lin28a,* and *Cbx7* (late pluripotency markers) was not upregulated when reprogramming with OSKB at MOI 2 compared to OSK (Figure 3E). We also found that the expression of early pluripotency markers such as *Fgf4*, *Etv5,* and *Sall4* was impaired when reprogramming with the OSKB lentivirus at MOI 2. Moreover, in these settings we failed to detect the expression of *Cdh1*, a gene that is normally upregulated during MET (Figure 3E). When we increased the number of lentiviral particles used for the transduction by 10-fold, we were partially able to rescue the reprogramming defect. Thus, the mRNA level of most of the genes analyzed had increased expression at this higher MOI, although still at lower expression levels when compared to cells reprogrammed with the OSK virus (Figure 3F). We also showed that the fold change in *Mybl2* expression levels decreased in OSKB compared to OSK as reprogramming progressed, which is indicative of a higher *Mybl2* expression in the OSK sample during reprogramming (Figure S4B). Moreover, after excision of the OSKB viral genomic DNA by Cre expression, we could see that iPSCs derived from OSKB MOI 20 showed

(B and C) Alkaline phosphatase staining (red) of Oct4-GFP MEFs at day 14 after transduction with MOI 2 (B) or MOI 20 (C).

(D) Bar graph representing the number of ESC-like colonies based on morphology and the number of Oct4-GFP-expressing colonies. Error bars represent SEMs. n = 3 biological replicates.

(E) Bar graph representing the number of ESC-like colonies based on morphology and the number of Nanog-GFP-expressing colonies. Error bars represent SEMs. n = 3 biological replicates. $^*p < 0.05$, $^{**}p < 0.01$.

(F) Oct4-GFP MEFs were transduced with OSKB or OSK lentiviral particles at MOI 2 or MOI 20. After 3 days the cells were fixed and stained for β-galactosidase activity, a marker of senescent cells. Representative image from 20× magnification inverted bright-field microscope. For positive control, MEFs were cultured at 100% confluence for 10 days without passage. n = 2 biological replicates.

(G and H) Wild-type MEFs at day 3 during reprogramming using OSKM, OSK or OSKB at MOI 2 (G) or MOI 20 (H). RNA expression of p21 (left), p16 (middle), and p53 (right) was measured by qRT-PCR, normalized against the β_2-microglobulin housekeeping gene, and expressed relative to non-transduced MEFs. n = 3 biological replicates.

(I) Wild-type MEFs were transduced with OSKM, OSK, or OSKB at MOI 2 or MOI 20. After 3 days, MEFs were stained for genomic DNA fragmentation with the TUNEL assay. Representative merged images at 40× magnification from confocal microscope show apoptotic nuclei (red) and DAPI (blue). Scale bar represents 50 μm. Positive control, MEFs treated with DNase I; negative control, non-transduced MEFs.

(J and K). Quantification of apoptotic nuclei counts from each condition MOI 2 (J) and MOI 20 (K). n = 2 biological replicates.

See also Figure S2.

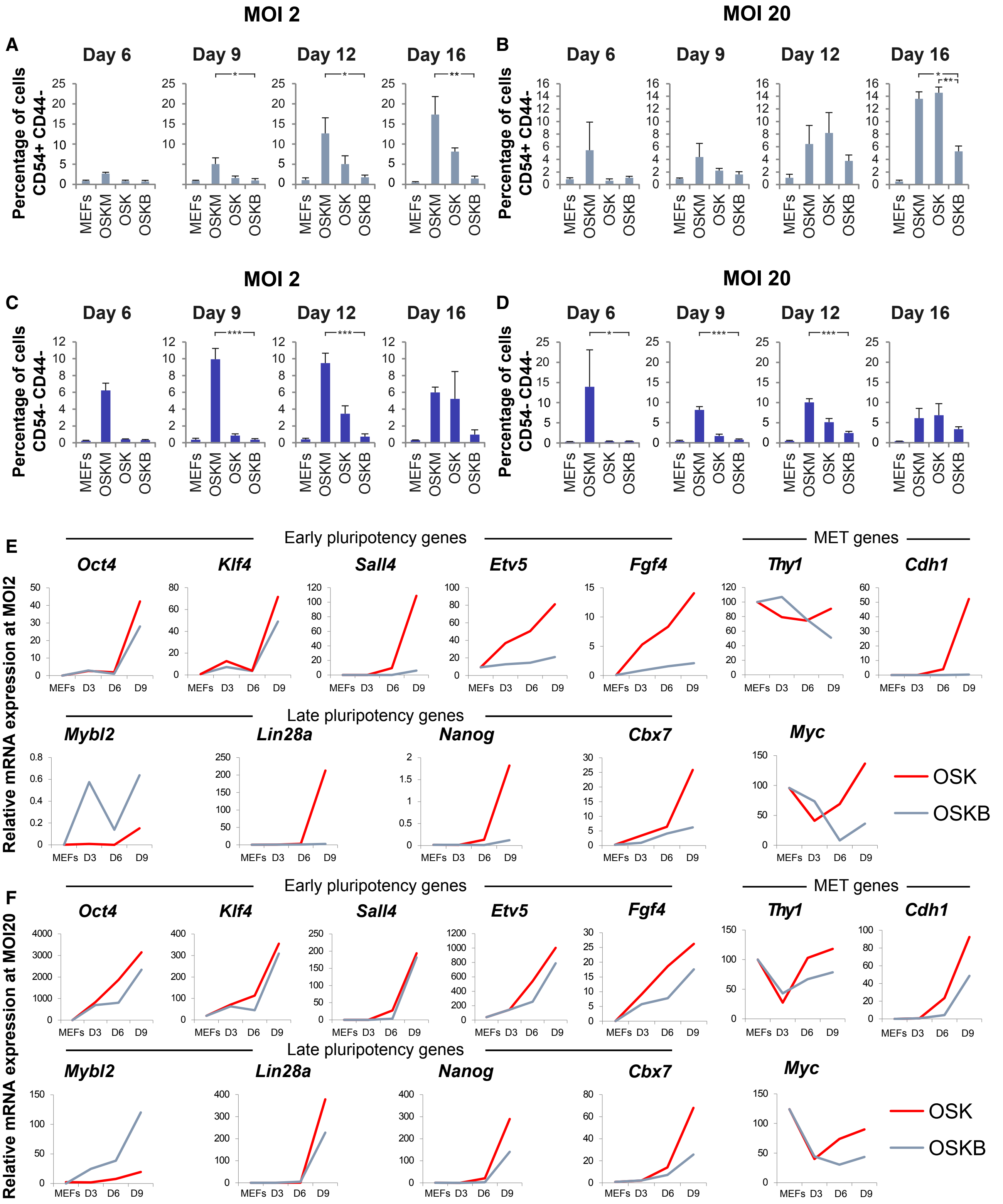

Figure 3. Reprogramming Kinetics in MEFs with Mybl2 Overexpression

(A–D) MEFs were transduced with OSK, OSKM, or OSKB lentiviral particles at MOI 2 (A and C) or MOI 20 (B and D) and then cultured up to 16 days. At days 6, 9, 12, and 16, MEFs were stained with CD44-APC and CD54-PE-Cy7 antibodies, and reprogramming kinetics were analyzed by flow cytometry. Graphs represent the percentage of $CD44^{-}/CD54^{+}$ or of $CD44^{-}/CD54^{-}$ cells. Error bars represent SEMs. n = 3 biological replicates. *p < 0.05, **p < 0.01, ***p < 0.001.

(legend continued on next page)

similar mRNA expression levels of the pluripotent genes to ESCs (Figure S4C).

Reprogramming Kinetics Are Affected in Transgenic Reprogrammable MEFs when Mybl2 Is Overexpressed

It has been reported that several reprogramming systems, including the STEMCCA vector that we used, have a short *Klf4* form, missing the first nine amino acids of wild-type *Klf4* (Chantzoura et al., 2015). To determine whether this could be the reason for the lack of reprogramming observed when Mybl2 was overexpressed, we used a reprogramming system with transgenic (Tg) MEFs carrying dox-inducible Yamanaka factors. These reprogrammable c-Myc, Klf4, Oct4, and Sox2 (MKOS) MEFs contain the four Yamanaka factors (with a full-length *Klf4*) integrated into the genome under the control of a tetracycline response element and an mOrange reporter of transgene expression (Chantzoura et al., 2015). A total of 10% MKOS MEFs was mixed with 90% wild-type (WT) MEFs and then transduced with Mybl2-AmCyan or AmCyan lentiviral particles. Reprogramming was initiated by the addition of dox, and reprogramming kinetics analyzing the expression of CD44 and CD54 were measured at days 9, 12, and 16 (Figures 4A and S5).

The mean percentage of $CD44^{-}/CD54^{+}$ cells when transduced with Mybl2 lentiviral particles was lower at days 9 and 12 compared to the AmCyan control. Furthermore, a greater difference was observed at day 16, with the percentage of those cells being significantly lower in Mybl2-transduced MEFs (6.79% ± 4.43%) compared to the corresponding AmCyan control (47.17% ± 17.43%) or untransduced cells (58% ± 4.8%, $p = 0.02$ and 0.002, respectively; Figure 4B). The percentage of pre-iPSCs ($CD44^{-}/CD54^{-}$) at days 12 and 16 was quite variable between experiments (Figure S6A), although at day 9, Mybl2 pre-iPS cells displayed a lower percentage of $CD44^{-}/CD54^{-}$ surface expression (1.08% ± 0.58%) when compared to the AmCyan control (8.96% ± 1.19%; Figure 4C). These data confirm that Mybl2 overexpression has a negative impact on the reprogramming efficiency. As before, we corroborated that Mybl2 overexpression did not have an impact on apoptosis or senescence during Tg MEF reprogramming by measuring the percentage of apoptotic nuclei and the expression of senescence-associated genes 3 days after dox induction (Figures 4D and 4E).

Less Detrimental Effect of Mybl2 Overexpression after Day 3 of Reprogramming

Having defined that Mybl2 has a negative impact on reprogramming, we next investigated the effects of Mybl2 overexpression at different time points of reprogramming. We transduced MKOS MEFs with AmCyan control or Mybl2-AmCyan lentiviral particles 3 days after the initiation of reprogramming (Figure S6B) and assessed the kinetics of reprogramming by analyzing the expression of CD44 and CD54 at days 9, 12, and 16, as indicated in Figure 4F. At day 9, a small percentage of $CD44^{-}/CD54^{+}$ iPSCs was observed in the non-transduced MKOS only (2.85% ± 1.57%), while both AmCyan and Mybl2-AmCyan MEFs displayed basal levels that were comparable to those of non-induced MEFs (Figure 4G). However, we could observe the appearance of partially reprogrammed $CD44^{-}/CD54^{-}$ cells in both AmCyan (6.07% ± 3.37%) and Mybl2-AmCyan MEFs (9.47% ± 4.27%) from day 9 (Figures 4H and S6C), unlike when the lentiviral transduction was performed on day −1 of reprogramming (Figure 4C). A large increase in the percentage of $CD44^{-}/CD54^{+}$ cells was observed at day 12 in all three conditions, being 32.83% ± 8.67% in the non-transduced, 27.07% ± 6.38% in the AmCyan-transduced, and 16.87% ± 1% in the Mybl2-AmCyan-transduced MKOS MEFs, maintaining the similar percentage until day 16 (Figure 4G). This represents a clear improvement in the efficiency of reprogramming when Mybl2 was overexpressed from day 3 in contrast to day −1 of reprogramming (Figure 4B). When MEFs were transduced with AmCyan control or Mybl2-AmCyan lentiviral particles at day 7 of reprogramming (Figure S6D), both AmCyan- and Mybl2-AmCyan-transduced MEFs displayed a great reduction in iPSCs formation (4% ± 1% and 8.4% ± 1%, respectively) compared to the non-transduced MEFs (53% ± 9%) (Figure S6E). This indicated that lentiviral infection at this time point was detrimental to efficient reprogramming and hindered precise assessment of the impact of Mybl2 overexpression. Nevertheless, the fact that Mybl2-overexpressing cells were reprogrammed equally well, or even slightly better, compared to AmCyan-expressing control cells indicated that the overexpression of Mybl2 from day 7 was not disadvantageous. In summary, these data clearly indicate that overexpressing Mybl2 at early time points can have a larger detrimental effect on reprogramming.

Mybl2 Overexpression on Transgenic Reprogrammable MEFs Leads to a Less Accessible Chromatin Conformation

In an attempt to precisely determine the mechanism underlying the detrimental nature of Mybl2 overexpression, we assessed the chromatin landscape by assay for transposase-accessible chromatin using sequencing (ATAC-seq) 3 days after the initiation of reprogramming. To do this, we transduced Tg reprogrammable MEFs with Mybl2-AmCyan or AmCyan control lentivirus 1 day before the initiation of reprogramming, and 3 days after dox addition (day 3) we sorted double-positive ($mOrange^{+}$ $AmCyan^{+}$) cells undergoing reprogramming and transduced with lentivirus. Finally, we used ATAC-seq (Buenrostro et al., 2015) to determine the open regions of chromatin (Figure 5A). For additional controls, we performed ATAC-seq in Tg reprogrammable MEFs at time zero and 4 days after Tg reprogrammable MEFs were transduced with Mybl2-AmCyan without going through the reprogramming process (no dox addition) (Figure 5A). This analysis revealed differentially regulated regions during reprogramming when Mybl2 was overexpressed (Figure 5B), with chromatin regions remaining closed when Mybl2 was overexpressed during reprogramming (black box at bottom

(E and F) Wild-type MEFs were reprogrammed with OSKB or OSK lentiviral particles at MOI 2 (E) or MOI 20 (F). At days 3, 6, or 9, expression of the indicated gene was measured using qRT-PCR normalized against the β_2-microglobulin gene and then expressed relatively to WT ESCs expression or MEFs (*Thy1* only). One representative graph is shown; n = 3 biological replicates, each with 3 technical replicates.
See also Figures S3 and S4.

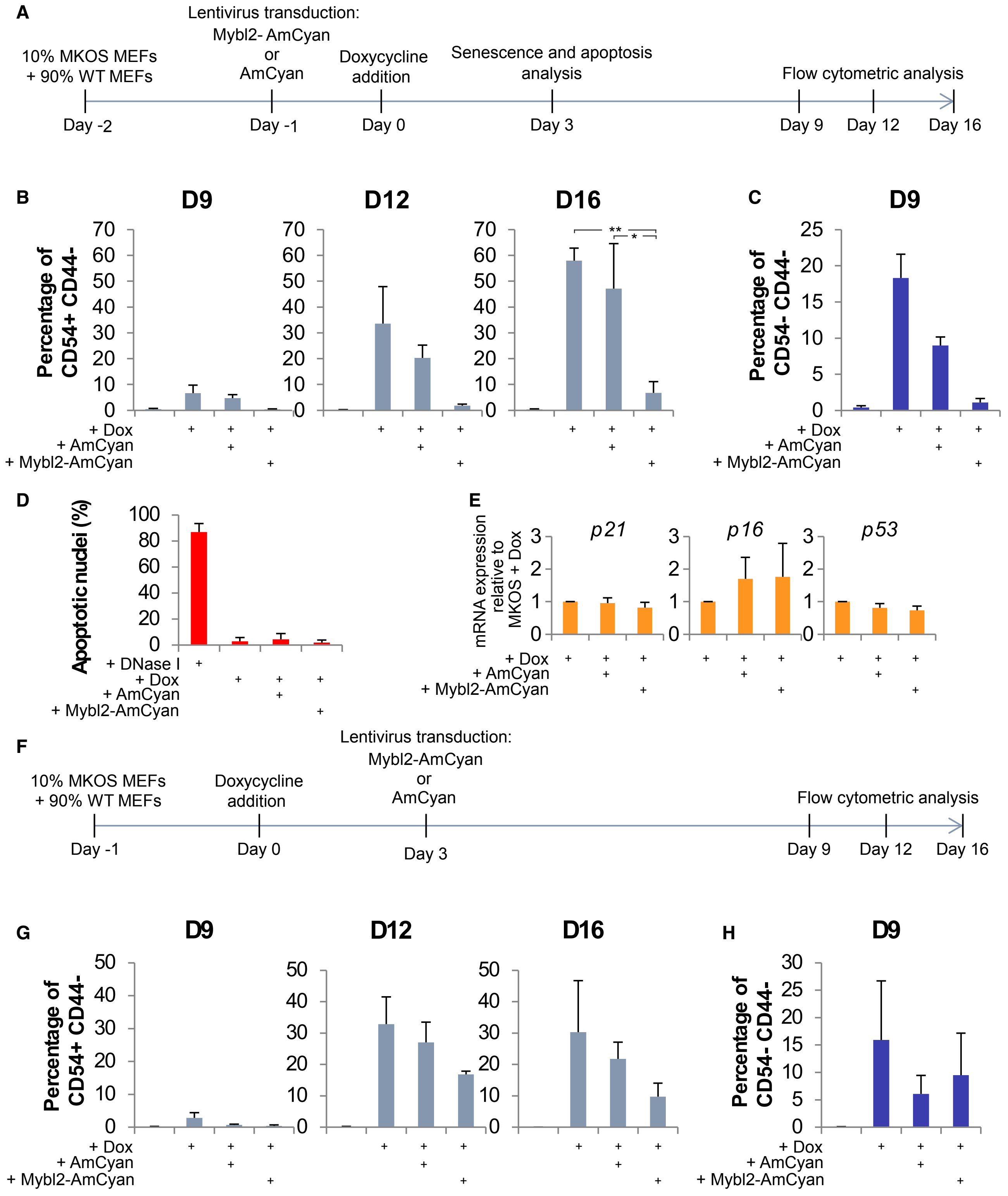

Figure 4. Mybl2 Overexpression Inhibits Tg Reprogrammable MEFs

(A) Schematic representation of the experimental design.

(B and C) Reprogramming kinetics studies using CD44-APC and CD54-PE-Cy7 at days 9, 12, and 16. Bar graphs represent the percentage of cells in the $CD44^-$/$CD54^+$ (B) or the $CD44^-$/$CD54^-$ quadrant (C). n = 3. $^*p < 0.05$, $^{**}p < 0.01$.

(legend continued on next page)

of Figure 5B) and chromatin regions remaining open (black box at top of Figure 5B). Because Yamanaka factors can act as pioneer factors (Knaupp et al., 2017; Soufi et al., 2012), we decided to determine whether the overexpression of Mybl2 could be affecting their binding to chromatin. We found it interesting that when ATAC-seq peaks were analyzed based on Oct4, Sox2, Oct4-Sox2, and Klf4 motifs, we observed an overall difference in Sox2 motifs when comparing control and Mybl2-AmCyan-transduced MKOS MEFs (Figure 5C). When the analysis was done on the ATAC-seq peaks that were different between both conditions (top 10% differentially open loci), a clear difference with respect to Oct4-Sox2, Sox2, and Oct4 motifs was observed between control and Mybl2-AmCyan-transduced MKOS (Figure 5D), suggesting that the overexpression of Mybl2 affects the binding of Sox2 and Oct4 to specific chromatin regions. In all cases, Oct4 and Sox2 motifs were not centered on the ATAC-seq peak, while Klf4 motifs were centered. Moreover, the analysis of the enriched motifs for the 10% of the peaks showing higher open chromatin in AmCyan control MKOS (black box at the bottom of Figure 5B) revealed that these regions were enriched in Klf4, Sox2, and Oct4/Sox2/Tcf./Nanog binding sites (Figure S7A). Conversely, the enriched motif analysis for the 10% of the peaks showing higher open chromatin levels in Mybl2-AmCyan MKOS (black box at the top of Figure 5B) revealed that these regions were enriched in AP1 sites (Fra1, Fosl2, and Jun-AP1 binding sites) (Figure S7B). These data suggest a less accessible environment for the binding of the Yamanaka factors on chromatin as a consequence of Mybl2 overexpression at this stage of the reprogramming. Furthermore, we took a closer look at unique peaks to determine whether Mybl2 MKOS would still display an enrichment in those specific motifs relative to the AmCyan control. A total of 377 unique ATAC-seq peaks were present in Mybl2-AmCyan control and 1,952 unique peaks were observed in the AmCyan corresponding control (Figure 5E). This analysis revealed that the unique peaks present in AmCyan preferentially displayed Sox2, Klf4, and Oct4-Sox2-Tcf.-Nanog motifs (Figure 5F), while AP1 motifs were enriched in those peaks that were uniquely observed in Mybl2-AmCyan-transduced MKOS (Figure 5G). Gene Ontology (GO) analysis of the genes associated with the peaks that were uniquely observed in AmCyan-transduced MKOS showed that the genes related to pluripotency were among the top 10 classes ($-\log P = 9.438$ compared with $-\log P = 1.648$ for Mybl2-AmCyan specific peaks; Figure 5H). Consistent with AP1 motifs being enriched in the peaks uniquely observed in Mybl2-AmCyan-transduced MKOS, the GO analysis revealed the mitogen-activated protein kinase (MAPK) signaling pathway as one of the top 10 enriched classes in Mybl2 MKOS ($-\log P = 5.969$ compared with $-\log P = 0.76$ for AmCyan-specific peaks; Figure 5I).

By intersecting our data with publicly available Jun and JunD chromatin immunoprecipitation sequencing (ChIP-seq) in MEFs and during somatic reprogramming (Liu et al., 2015; Vierbuchen et al., 2017), we observed that AP1 is binding in Mybl2-specific ATAC peaks and shared peaks in WT MEFs and *Klf4*, *Sox2*, *Myc* (KSM) MEFs (Figure S7C). Similarly, the intersection of our ATAC-seq data with publicly available ChIP-seq data for Oct4, Sox2, and Nanog in ESCs (Whyte et al., 2013), iPSCs, and MEFs at day 3 of reprogramming (Knaupp et al., 2017) revealed that MEFs at day 3 of reprogramming, iPSCs, and mouse ESC (mESC) profiles (especially Sox2) were very similar to those observed in the AmCyan ATAC-seq (Figures S7D and S7E).

The presence of AP1 motifs in the Mybl2-AmCyan- but not in AmCyan-specific peaks also hints at the presence of active somatic enhancers, which are normally repressed during efficient reprogramming (Chronis et al., 2017). Taken together, these data demonstrate the detrimental effect of Mybl2 overexpression during somatic reprogramming, the biological consequence of which is reflected by a less permissive chromatin landscape for the binding of Yamanaka factors to their specific target regions and a more permissive chromatin landscape for the binding of early immediate response genes that are known to be reprogramming blockers (Li et al., 2017; Liu et al., 2015).

Mybl2 Overexpression during Reprogramming Deregulates Mesenchymal-to-Epithelial Transition

These observations prompted us to determine whether the changes in chromatin accessibility would lead to a distinct molecular signature and a specific transcriptome associated with impaired reprogramming. Thus, we set out to perform global gene expression profiling by RNA-seq comparing Tg reprogrammable MEFs transduced either with Mybl2-AmCyan or AmCyan control 3 days after the initiation of reprogramming. With this approach, we identified 350 differentially expressed genes (Figures 6A and 6B); these genes were mostly implicated in the regulation of cell adhesion and extracellular matrix organization GO categories such as *Postn, Col3a1, Col5a2, Col11a1*, *Adamts5,* and *Fmod* (Figures 6C and S8A). Gene set enrichment analysis (GSEA) revealed a negative correlation with the expression of the genes that are part of the epithelial-to-mesenchymal transition pathway (MSigDB ref: M5930) (nominal [NOM] p value <0.001, family-wise error rate [FWER] p value <0.001, as shown in Figure 6D). Contrary to our expectations, these data seem to suggest that Mybl2 expression is promoting a rapid downregulation of the mesenchymal genes, one of the first steps for somatic reprogramming to occur. Nonetheless, the key genes required for proper MET transition and establishment of an epithelial fate were deregulated, such as the epithelial genes *Cdh1, Icam1, Celsr2, Krt8,* and *Krt80* and the mesenchymal gene *Lef1*. Cross-linking-ChIP (X-ChIP) analysis on immortalized

(D) Quantification of apoptotic nuclei counts at day 3 or reprogramming. n = 2 biological replicates.

(E) RNA expression of *p21* (left), *p16* (middle), and *p53* (right) was measured by qRT-PCR, normalized against the β_2-*microglobulin* housekeeping gene and expressed relative to non-transduced Tg reprogrammable MEFs (MKOS). n = 3 biological replicates, each with 3 technical replicates.

(F) Schematic representation of the experimental design.

(G and H) Graphs represent the percentage of $CD44^{-}/CD54^{+}$ cells (G) at days 9, 12, and 16 or the percentage of $CD44^{-}/CD54^{-}$ cells (H) at day 9. n = 3 biological replicates.

See also Figures S5 and S6.

OPEN ACCESS
CellPress

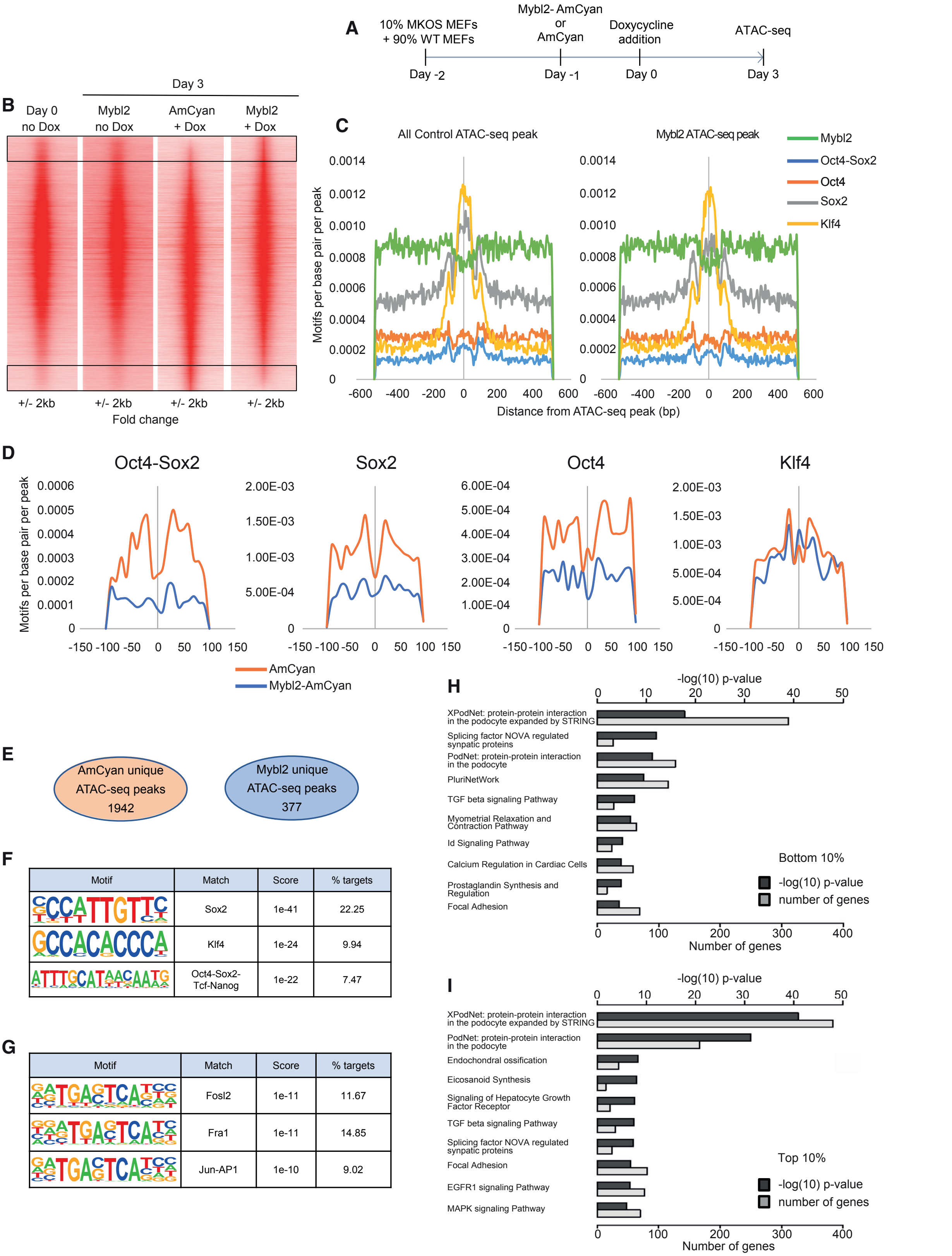

(legend on next page)

MEFs corroborated the binding of Mybl2 to *Lef1, Tgfbr3, Col11a1,* and *Col6a2* (Figure S8B). These data suggest that Mybl2 overexpression during reprogramming alters the proper cell fate decisions required for the MET transition that takes place in the initiation steps of somatic reprogramming (Figures 6E, 6F, S8C, and S8D).

In conclusion, these data demonstrate that high levels of Mybl2 during the early stages of reprogramming affect the chain of events that are required for reprogramming to occur and thus suggest that overexpression of Mybl2 at an early time point blocks the reprogramming process by affecting the chromatin landscape, thereby deregulating the expression of key genes such as *Lef1* and *Cdh1* that are required for proper MET transition (Figure 7).

DISCUSSION

In this study, we found an important and as-yet uncharacterized function for Mybl2 in somatic cellular reprogramming that highlights the importance of both the timing and the levels of Mybl2 expression. As expected, given the critical role of Mybl2 in proliferation (Musa et al., 2017), cells that did not express Mybl2 were incapable of reprogramming. We set out to study how an increase in the expression of Mybl2 could affect somatic reprogramming using either a single polycistronic vector or secondary reprogramming MEFs. We found that coexpression of Mybl2 together with Oct4, Sox2, and Klf4 profoundly impaired the efficiency of reprogramming. These results are in clear contrast to other published work in which important genes for reprogramming have been identified, resulting in opposite effects when loss-of-function and gain-of-function experiments were performed (Banito and Gil, 2010; Chen et al., 2013; Hanna et al., 2009; Kawamura et al., 2009; Maekawa et al., 2011; Onder et al., 2012; Silva et al., 2009; Unternaehrer et al., 2014).

We confirmed that the exogenous pluripotent genes were expressed and that the cells infected with OSKB or Tg reprogrammable MEFs infected with Mybl2-AmCyan had not become senescent or apoptotic, two important barriers for the reprogramming process (Banito and Gil, 2010; Hong et al., 2009; Marión et al., 2009; Utikal et al., 2009). Moreover, a time course of MEFs infected with either OSKB or Tg reprogrammable MEFs infected with Mybl2-AmCyan demonstrated the absence of physical characteristics associated with reprogramming, because those cells did not exhibit the expected kinetics of CD44/CD54 expression (O'Malley et al., 2013). We also observed that the upregulation of early and late pluripotency genes was hampered when cells were infected with OSKB or Tg reprogrammable MEFs infected with Mybl2-AmCyan (Figure 3), indicating that increasing Mybl2 levels unbalance the molecular events required for the early stages of reprogramming.

Different studies have shown a specific roadmap during the reprogramming process, with two waves during reprogramming that encompass three different steps: initiation, maturation, and stabilization (Polo et al., 2012; Samavarchi-Tehrani et al., 2010). It has also become increasingly clear that during the initiation phase, cell fate decisions change from a mesenchymal to an epithelial program. Hence, fibroblasts downregulate mesenchymal-related genes to suppress the epithelial-to-mesenchymal transition (EMT) pathway with a concomitant activation of the MET, upregulating the expression of epithelial genes, with *E-cadherin* being one of the key genes for the establishment of the epithelial fate (Li et al., 2010).

Suppression of the EMT during the initiation of reprogramming requires the inhibition of transforming growth factor-β (TGF-β) signaling by the Yamanaka factors (Li et al., 2010). Our RNA-seq data show that Mybl2-AmCyan-transduced Tg reprogrammable MEFs rapidly downregulate genes (e.g., *TGFbr3*) that are important for the suppression of the EMT and a rapid downregulation of mesenchymal genes (e.g., components of the extracellular matrix such as the collagen family of proteins) (Li et al., 2010; Samavarchi-Tehrani et al., 2010). Our data also showed that overexpression of Mybl2 in Tg reprogrammable MEFs changes the chromatin landscape of the cell, leading to changes in open regions on genes implicated in the regulation of the MET transition. Nonetheless, other crucial factors for the establishment of the MET are deregulated. Thus, Mybl2-AmCyan-transduced Tg reprogrammable MEFs are unable to downregulate the T cell factor *Lef1*, which belongs to a family of transcription factors that modulates the transcription of genes by recruiting chromatin remodeling and histone-modifying complexes to their target genes (Hoppler and Kavanagh, 2007; Merrill et al., 2001). *Lef1* has been shown to have a key role in inhibiting the early stages of reprogramming by activating Wnt signaling.

Figure 5. Mybl2 Overexpression Changes the Chromatin Landscape in the Initiation of Reprogramming of Tg Reprogrammable MEFs

(A) Schematic representation of the experimental design.

(B) Profiles of the ATAC-seq signals within each 1,000-bp window centered on each peak for Tg MEFs at time zero (day 0 no dox) (n = 2 biological replicates), Tg MEFs 4 days after Mybl2-AmCyan infection, no reprogramming (Mybl2 no dox) (n = 2 biological replicates), and Tg MEFs infected with AmCyan or Mybl2-AmCyan and 3 days after dox addition (AmCyan + dox, Mybl2-AmCyan + dox, respectively) (n = 3 biological replicates). Peaks are shown in the order of decreasing ATAC-seq tag count signal for Mybl2-treated relative to control (AmCyan).

(C) Reprogramming pioneer factor and Mybl2 motif density within each 200-bp window centered on each peak in all control ATAC-seq peaks compared to all Mybl2 ATAC-seq peaks.

(D) Reprogramming pioneer factor motif density in top 10% (blue) or bottom 10% ATAC-seq peaks (orange) from the union of peaks between control and Mybl2 overexpressed cells at day 3 of reprogramming.

(E) Number of unique ATAC-seq peaks in control and treated cells after performing differential peak detection.

(F) Motif enrichment analysis in unique control ATAC-seq peaks at day 3 of reprogramming.

(G) Motif enrichment analysis in Mybl2-AmCyan ATAC-seq peaks at day 3 of reprogramming.

(H) Gene Ontology (GO) analysis for the closest gene to unique ATAC-seq peaks present in AmCyan control at day 3 of reprogramming (bottom 10%).

(I) GO analysis for the closest gene to unique ATAC-seq peaks present in Mybl2-AmCyan at day 3 of reprogramming (top 10%).

See also Figure S7.

OPEN ACCESS
CellPress

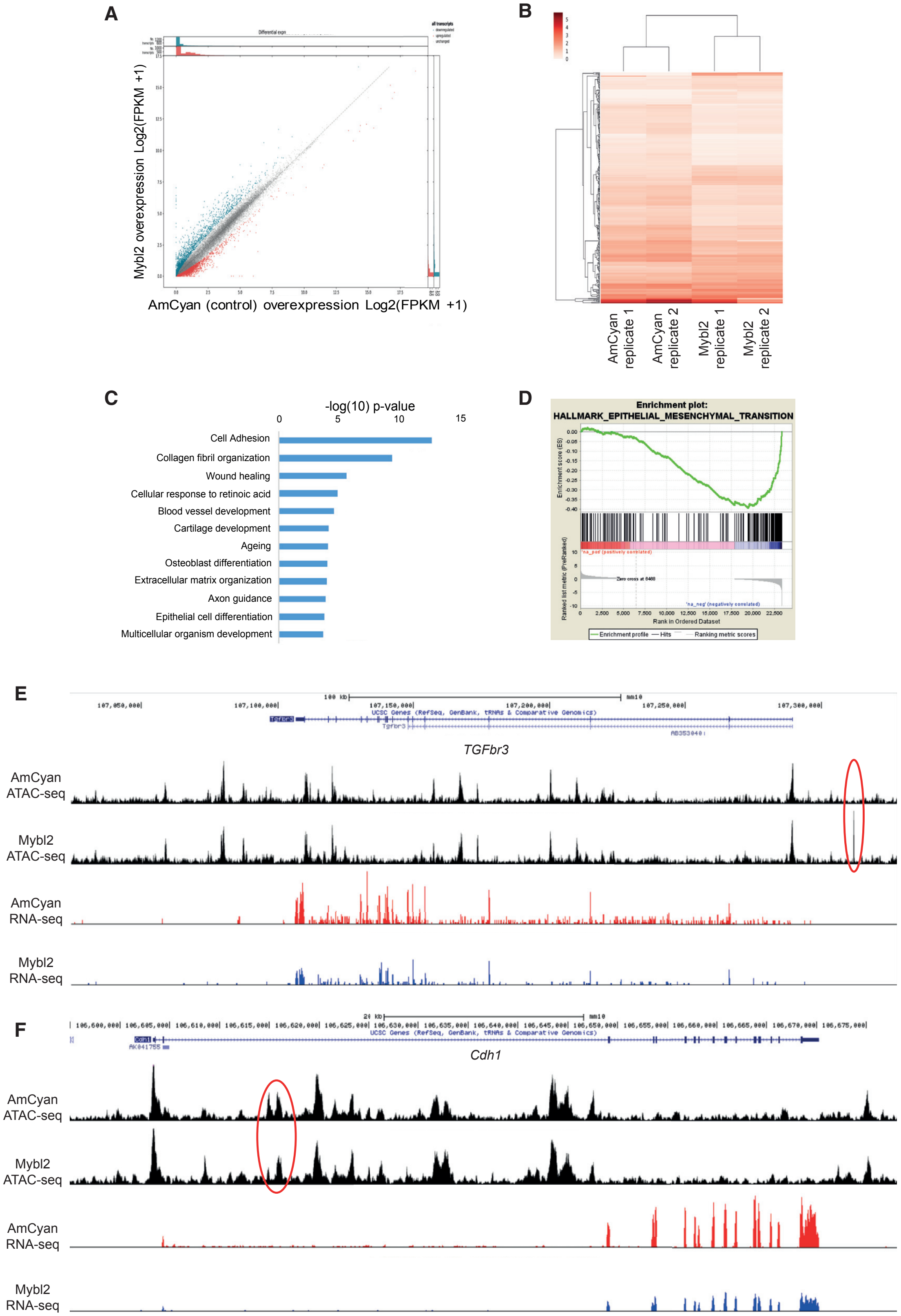

(legend on next page)

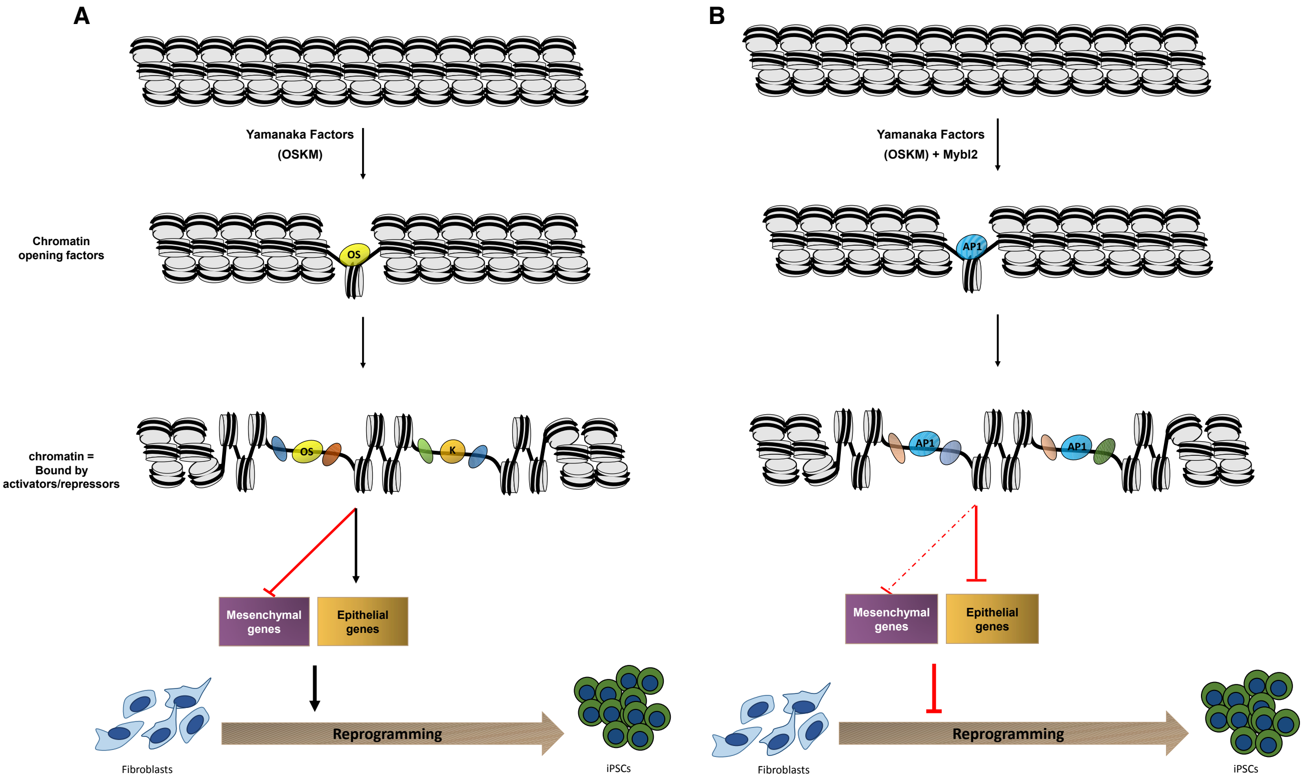

Figure 7. Molecular Network for the Deregulation of Cell Fate Decision Driven by Mybl2 Overexpression during Somatic Reprogramming

(A) Schematic representation of the mechanism by which Yamanaka factors Oct4 (O), Sox2 (S), and Klf4 (K) reprogram somatic cells by opening chromatin regions and regulating specific genes required to promote the mesenchymal-to-epithelial transition (MET).

(B) Schematic representation showing chromatin landscape changes in the presence of overexpressed Mybl2. Here, regions of open chromatin are bound by AP1 transcriptions factors. This binding deregulates genes important for the crucial mesenchymal-to-epithelial transition phase occurring during somatic reprogramming.

Accordingly, its loss of function increases the number of Nanog$^+$ colonies during somatic reprograming (Ho et al., 2013); hence, the lack of reprogramming observed when Mybl2 is overexpressed at the same time that the OSK factors could be the result of a failure to downregulate *Lef1*.

Our data also showed a failure in the upregulation of epithelial markers that are required for the initiation of the reprogramming process in Tg reprogrammable MEFs infected with Mybl2-AmCyan; among these are key genes required for the MET, such as *E-cadherin* (*Cdh1*). E-cadherin, a regulator of epithelial homeostasis (Cavallaro and Christofori, 2004), has been shown to be required for reprogramming (Li et al., 2010) because its downregulation by small hairpin RNAs (shRNAs) (Li et al., 2010) or small interfering RNAs (siRNAs) (Samavarchi-Tehrani et al., 2010) reduces the number of iPSC colonies generated, while its overexpression enhances the generation of iPSCs (Chen et al., 2010). We also observed changes in the chromatin landscape surrounding the *Cdh1* locus when Mybl2 was overexpressed.

Our genome-wide data showed that the overexpression of Mybl2 during reprogramming affects the way chromatin is

Figure 6. Deregulation of Genes Important for Cell Fate Decision when Mybl2 Is Overexpressed in Tg Reprogrammable MEFs

(A) Scatterplot showing RNA-seq expression of all detected genes in AmCyan control cells versus Mybl2 overexpressing Tg reprogrammable MEFs after 3 days of reprogramming. Points are blue where fold change was >2 in Mybl2 or red where fold change was >2 in AmCyan.

(B) Expression heatmap of significantly different genes between control and Mybl2 overexpressing Tg reprogrammable MEFs at day 3 of reprogramming. Genes and samples were grouped using average linkage hierarchical clustering. The dendrogram shows groups of genes that clustered together.

(C) GO analysis of significantly different genes from RNA-seq analysis. The genes were checked for biological functions using Database for Annotation, Visualization, and Integrated Discovery.

(D) Gene set enrichment analysis of RNA-seq genes ranked based on significance and tested against Hallmark gene molecular signature database.

(E) ATAC-seq and RNA-seq University of California, Santa Cruz (UCSC) genome browser tracks for *Tgfbr3*, the downregulation of which is required for somatic reprogramming. Red ovals show differential ATAC-seq peak between both conditions containing AP1 motives.

(F) ATAC-seq and RNA-seq UCSC genome browser tracks for deregulated epithelial gene *Cdh1*. Red ovals show differential ATAC-seq peak between both conditions containing Klf4 and Sox2 motives.

See also Figure S8.

remodeled and the binding of pioneer factors to specific regions. Thus, nearly 2,000 ATAC-seq peaks, 40% of them enriched in Yamanaka factor motifs, were absent when Mybl2 was overexpressed in Tg reprogrammable MEFs. These regions were closed in MEFs at time zero and were enriched on Sox2, Klf4, and Oct4 motifs by day 3 in the AmCyan control. Thus, contrary to recent reports in which OSK was found to mainly target open chromatin sites in MEFs (Chen et al., 2016; Chronis et al., 2017), our work supports the hypothesis that Sox2, Klf4, and Oct4 act as pioneer factors during reprogramming, which is in agreement with other studies (Knaupp et al., 2017; Li et al., 2017; Soufi et al., 2012). At the same time, since we observed that OSK was not only present in closed chromatin but also in shared, open regions, both standpoints can be reconciled to hold true. Moreover, our data suggest that certain regions fail to close upon OKSM induction because of Mybl2 overexpression and that Mybl2 favors the binding of early immediate response genes from the AP1 family, which is known to be reprogramming blockers (Li et al., 2017; Liu et al., 2015); however, the direct binding of Mybl2 to those sites during somatic reprogramming still needs to be validated.

Overall, our data points to Mybl2 as a gatekeeper for somatic reprogramming, the levels of which are important for maintaining a chromatin landscape that will favor the binding of pioneer factors over reprogramming blockers. This, therefore, regulates the switch fate that fibroblasts are subjected to during the initiation stage; that is, the concomitant loss of their mesenchymal signature and gain of an epithelial identity.

STAR★METHODS

Detailed methods are provided in the online version of this paper and include the following:

- KEY RESOURCES TABLE
- CONTACT FOR REAGENTS AND RESOURCE SHARING
- EXPERIMENTAL MODEL AND SUBJECT DETAILS
  - Cell lines and primary cells
  - Mice used for the generation of MEFs
- METHOD DETAILS
  - Lentiviral production
  - Primary reprogramming
  - Somatic reprogramming using Tg-reprogrammable MEFs
  - Flow cytometric analysis of reprogramming process
  - Generation of pHAGE-2-EF1a-mOSKB, pHAGE-2-EF1a-AmCyan, pHAGE-2-EF1a-Mybl2-HA-P2A-EF1a-AmCyan
  - Generation of Fu-TetO-Cre-P2A-ZsGreen
  - Gibson Assembly
  - Gateway cloning
  - Cell Sorting
  - Quantitative RT-PCR determination of mRNA levels
  - AP staining
  - Senescence staining
  - Apoptosis staining (TUNEL)
  - ATAC-seq
  - Alignment of ATAC-seq data
  - Peak detection and filtering; coverage track generation
  - Two-way fold change analysis
  - Motif discovery and motif density plots
  - RNA-seq
  - Gene set enrichment analysis
  - Chromatin Immunoprecipitation
- QUANTIFICATION AND STATISTICAL ANALYSIS
- DATA AND SOFTWARE AVAILABILITY

SUPPLEMENTAL INFORMATION

Supplemental Information includes eight figures and can be found with this article online at https://doi.org/10.1016/j.celrep.2018.07.026.

ACKNOWLEDGMENTS

The authors wish to thank the members of the Garcia group for advice and constructive criticisms, Professor Bonifer for advice and critical reading, Drs. Sarah Beavington and Peter Keane for advice on RNA-seq and ATAC-seq libraries and bioinformatic analysis, Dr. Jorge Cammano for the *p53*$^{-/-}$ mice, and Professor Cockerill for the chromatin illustrations. The authors also wish to thank the Biomedical Services Unit for their assistance with the animal studies. FU-TetO-Gateway plasmid was a gift from John Gearhart (Addgene plasmid 43914). C.W. was supported by an MRC Research Capacity Building PhD studentship (1092244). K.K. is an MRC Centre for Regenerative Medicine senior non-clinical fellow MR/N008715/1. This work was also funded by a Wellcome Trust Institutional Strategic Support Fund (ISSF) mobility training grant (097825/Z/11/B) to C.W. and 097825/Z/11/A, 097825/Z/11/B Wellcome Trust ISSF grant to P.G.

AUTHOR CONTRIBUTIONS

C.W. conceived and performed the experiments, acquired and analyzed the data, analyzed the RNA-seq and ATAC-seq data, and wrote the manuscript. G.V. performed the experiments, acquired and analyzed the data, and contributed to the writing of the manuscript. A.P., R.A., D.B., M.N., and D.K. performed the experiments. P.C. analyzed the ATAC-seq data and edited the manuscript. G.M. and J.F. provided reagents and helped with the experimental design. Y.B. helped with the experimental design, interpretation, and critical discussion of the data and edited the manuscript. K.K. provided reagents and helped with the experimental design, interpretation, and critical discussion of the data and edited the manuscript. P.G. conceived and performed the experiments, acquired, analyzed, and interpreted the data, wrote the manuscript, and managed the project.

DECLARATION OF INTERESTS

The authors declare no competing interests.

Received: December 20, 2017
Revised: May 18, 2018
Accepted: July 6, 2018
Published: August 7, 2018

REFERENCES

Addis, R.C., Hsu, F.C., Wright, R.L., Dichter, M.A., Coulter, D.A., and Gearhart, J.D. (2011). Efficient conversion of astrocytes to functional midbrain dopaminergic neurons using a single polycistronic vector. PLoS One *6*, e28719.

Ahlbory, D., Appl, H., Lang, D., and Klempnauer, K.H. (2005). Disruption of B-myb in DT40 cells reveals novel function for B-Myb in the response to DNA-damage. Oncogene *24*, 7127–7134.

Banito, A., and Gil, J. (2010). Induced pluripotent stem cells and senescence: learning the biology to improve the technology. EMBO Rep. *11*, 353–359.

Benchetrit, H., Herman, S., van Wietmarschen, N., Wu, T., Makedonski, K., Maoz, N., Yom Tov, N., Stave, D., Lasry, R., Zayat, V., et al. (2015). Extensive nuclear reprogramming underlies lineage conversion into functional trophoblast stem-like cells. Cell Stem Cell *17*, 543–556.

Brignall, R., Cauchy, P., Bevington, S.L., Gorman, B., Pisco, A.O., Bagnall, J., Boddington, C., Rowe, W., England, H., Rich, K., et al. (2017). Integration of kinase and calcium signaling at the level of chromatin underlies inducible gene activation in T cells. J. Immunol. *199*, 2652–2667.

Buenrostro, J.D., Wu, B., Chang, H.Y., and Greenleaf, W.J. (2015). ATAC-seq: a method for assaying chromatin accessibility genome-wide. Curr. Protoc. Mol. Biol. *109*, 21.29.1–21.29.9.

Buganim, Y., Faddah, D.A., Cheng, A.W., Itskovich, E., Markoulaki, S., Ganz, K., Klemm, S.L., van Oudenaarden, A., and Jaenisch, R. (2012). Single-cell expression analyses during cellular reprogramming reveal an early stochastic and a late hierarchic phase. Cell *150*, 1209–1222.

Cavallaro, U., and Christofori, G. (2004). Cell adhesion and signalling by cadherins and Ig-CAMs in cancer. Nat. Rev. Cancer *4*, 118–132.

Chambers, I., Silva, J., Colby, D., Nichols, J., Nijmeijer, B., Robertson, M., Vrana, J., Jones, K., Grotewold, L., and Smith, A. (2007). Nanog safeguards pluripotency and mediates germline development. Nature *450*, 1230–1234.

Chantzoura, E., Skylaki, S., Menendez, S., Kim, S.I., Johnsson, A., Linnarsson, S., Woltjen, K., Chambers, I., and Kaji, K. (2015). Reprogramming roadblocks are system dependent. Stem Cell Reports *5*, 350–364.

Chen, T., Yuan, D., Wei, B., Jiang, J., Kang, J., Ling, K., Gu, Y., Li, J., Xiao, L., and Pei, G. (2010). E-cadherin-mediated cell-cell contact is critical for induced pluripotent stem cell generation. Stem Cells *28*, 1315–1325.

Chen, J., Liu, H., Liu, J., Qi, J., Wei, B., Yang, J., Liang, H., Chen, Y., Chen, J., Wu, Y., et al. (2013). H3K9 methylation is a barrier during somatic cell reprogramming into iPSCs. Nat. Genet. *45*, 34–42.

Chen, J., Chen, X., Li, M., Liu, X., Gao, Y., Kou, X., Zhao, Y., Zheng, W., Zhang, X., Huo, Y., et al. (2016). Hierarchical Oct4 binding in concert with primed epigenetic rearrangements during somatic cell reprogramming. Cell Rep. *14*, 1540–1554.

Chronis, C., Fiziev, P., Papp, B., Butz, S., Bonora, G., Sabri, S., Ernst, J., and Plath, K. (2017). Cooperative binding of transcription factors orchestrates reprogramming. Cell *168*, 442–459.e20.

Fung, S.M., Ramsay, G., and Katzen, A.L. (2002). Mutations in Drosophila myb lead to centrosome amplification and genomic instability. Development *129*, 347–359.

García, P., and Frampton, J. (2006). The transcription factor B-Myb is essential for S-phase progression and genomic stability in diploid and polyploid megakaryocytes. J. Cell Sci. *119*, 1483–1493.

García, P., Berlanga, O., Watson, R., and Frampton, J. (2005). Generation of a conditional allele of the B-myb gene. Genesis *43*, 189–195.

Hanna, J., Saha, K., Pando, B., van Zon, J., Lengner, C.J., Creyghton, M.P., van Oudenaarden, A., and Jaenisch, R. (2009). Direct cell reprogramming is a stochastic process amenable to acceleration. Nature *462*, 595–601.

Hansson, J., Rafiee, M.R., Reiland, S., Polo, J.M., Gehring, J., Okawa, S., Huber, W., Hochedlinger, K., and Krijgsveld, J. (2012). Highly coordinated proteome dynamics during reprogramming of somatic cells to pluripotency. Cell Rep. *2*, 1579–1592.

Heinz, S., Benner, C., Spann, N., Bertolino, E., Lin, Y.C., Laslo, P., Cheng, J.X., Murre, C., Singh, H., and Glass, C.K. (2010). Simple combinations of lineage-determining transcription factors prime cis-regulatory elements required for macrophage and B cell identities. Mol. Cell *38*, 576–589.

Ho, R., Papp, B., Hoffman, J.A., Merrill, B.J., and Plath, K. (2013). Stage-specific regulation of reprogramming to induced pluripotent stem cells by Wnt signaling and T cell factor proteins. Cell Rep. *3*, 2113–2126.

Hong, H., Takahashi, K., Ichisaka, T., Aoi, T., Kanagawa, O., Nakagawa, M., Okita, K., and Yamanaka, S. (2009). Suppression of induced pluripotent stem cell generation by the p53-p21 pathway. Nature *460*, 1132–1135.

Hoppler, S., and Kavanagh, C.L. (2007). Wnt signalling: variety at the core. J. Cell Sci. *120*, 385–393.

Kawamura, T., Suzuki, J., Wang, Y.V., Menendez, S., Morera, L.B., Raya, A., Wahl, G.M., and Izpisúa Belmonte, J.C. (2009). Linking the p53 tumour suppressor pathway to somatic cell reprogramming. Nature *460*, 1140–1144.

Knaupp, A.S., Buckberry, S., Pflueger, J., Lim, S.M., Ford, E., Larcombe, M.R., Rossello, F.J., de Mendoza, A., Alaei, S., Firas, J., et al. (2017). Transient and permanent reconfiguration of chromatin and transcription factor occupancy drive reprogramming. Cell Stem Cell *21*, 834–845.e6.

Langmead, B., and Salzberg, S.L. (2012). Fast gapped-read alignment with Bowtie 2. Nat. Methods *9*, 357–359.

Lengner, C.J., Camargo, F.D., Hochedlinger, K., Welstead, G.G., Zaidi, S., Gokhale, S., Scholer, H.R., Tomilin, A., and Jaenisch, R. (2007). Oct4 expression is not required for mouse somatic stem cell self-renewal. Cell Stem Cell *1*, 403–415.

Li, R., Liang, J., Ni, S., Zhou, T., Qing, X., Li, H., He, W., Chen, J., Li, F., Zhuang, Q., et al. (2010). A mesenchymal-to-epithelial transition initiates and is required for the nuclear reprogramming of mouse fibroblasts. Cell Stem Cell *7*, 51–63.

Li, D., Liu, J., Yang, X., Zhou, C., Guo, J., Wu, C., Qin, Y., Guo, L., He, J., Yu, S., et al. (2017). Chromatin accessibility dynamics during iPSC reprogramming. Cell Stem Cell *21*, 819–833.e6.

Liu, J., Han, Q., Peng, T., Peng, M., Wei, B., Li, D., Wang, X., Yu, S., Yang, J., Cao, S., et al. (2015). The oncogene c-Jun impedes somatic cell reprogramming. Nat. Cell Biol. *17*, 856–867.

Lorvellac, M., Dumon, S., Maya-Mendoza, A., Jackson, D., Frampton, J., and Garcia, P. (2010). B-Myb is critical for proper DNA duplication during an unperturbed S phase in mouse embryonic stem cells. Stem Cells *28*, 1751–1759.

Maekawa, M., Yamaguchi, K., Nakamura, T., Shibukawa, R., Kodanaka, I., Ichisaka, T., Kawamura, Y., Mochizuki, H., Goshima, N., and Yamanaka, S. (2011). Direct reprogramming of somatic cells is promoted by maternal transcription factor Glis1. Nature *474*, 225–229.

Manak, J.R., Mitiku, N., and Lipsick, J.S. (2002). Mutation of the Drosophila homologue of the Myb protooncogene causes genomic instability. Proc. Natl. Acad. Sci. USA *99*, 7438–7443.

Marión, R.M., Strati, K., Li, H., Murga, M., Blanco, R., Ortega, S., Fernandez-Capetillo, O., Serrano, M., and Blasco, M.A. (2009). A p53-mediated DNA damage response limits reprogramming to ensure iPS cell genomic integrity. Nature *460*, 1149–1153.

Martin, M. (2011). Cutadapt removes adapter sequences from high-throughput sequencing reads. (2011). EMBnet J. *17*, 10–12.

Merrill, B.J., Gat, U., DasGupta, R., and Fuchs, E. (2001). Tcf3 and Lef1 regulate lineage differentiation of multipotent stem cells in skin. Genes Dev. *15*, 1688–1705.

Mostoslavsky, G., Fabian, A.J., Rooney, S., Alt, F.W., and Mulligan, R.C. (2006). Complete correction of murine Artemis immunodeficiency by lentiviral vector-mediated gene transfer. Proc. Natl. Acad. Sci. USA *103*, 16406–16411.

Musa, J., Aynaud, M.M., Mirabeau, O., Delattre, O., and Grünewald, T.G. (2017). MYBL2 (B-Myb): a central regulator of cell proliferation, cell survival and differentiation involved in tumorigenesis. Cell Death Dis. *8*, e2895.

O'Malley, J., Skylaki, S., Iwabuchi, K.A., Chantzoura, E., Ruetz, T., Johnsson, A., Tomlinson, S.R., Linnarsson, S., and Kaji, K. (2013). High-resolution analysis with novel cell-surface markers identifies routes to iPS cells. Nature *499*, 88–91.

Onder, T.T., Kara, N., Cherry, A., Sinha, A.U., Zhu, N., Bernt, K.M., Cahan, P., Marcarci, B.O., Unternaehrer, J., Gupta, P.B., et al. (2012). Chromatin-modifying enzymes as modulators of reprogramming. Nature *483*, 598–602.

Polo, J.M., Anderssen, E., Walsh, R.M., Schwarz, B.A., Nefzger, C.M., Lim, S.M., Borkent, M., Apostolou, E., Alaei, S., Cloutier, J., et al. (2012). A molecular roadmap of reprogramming somatic cells into iPS cells. Cell *151*, 1617–1632.

Quinlan, A.R., and Hall, I.M. (2010). BEDTools: a flexible suite of utilities for comparing genomic features. Bioinformatics *26*, 841–842.

Rafiee, M.R., Girardot, C., Sigismondo, G., and Krijgsveld, J. (2016). Expanding the circuitry of pluripotency by selective isolation of chromatin-associated proteins. Mol. Cell *64*, 624–635.

Samavarchi-Tehrani, P., Golipour, A., David, L., Sung, H.K., Beyer, T.A., Datti, A., Woltjen, K., Nagy, A., and Wrana, J.L. (2010). Functional genomics reveals a BMP-driven mesenchymal-to-epithelial transition in the initiation of somatic cell reprogramming. Cell Stem Cell *7*, 64–77.

Shepard, J.L., Amatruda, J.F., Stern, H.M., Subramanian, A., Finkelstein, D., Ziai, J., Finley, K.R., Pfaff, K.L., Hersey, C., Zhou, Y., et al. (2005). A zebrafish bmyb mutation causes genome instability and increased cancer susceptibility. Proc. Natl. Acad. Sci. USA *102*, 13194–13199.

Silva, J., Nichols, J., Theunissen, T.W., Guo, G., van Oosten, A.L., Barrandon, O., Wray, J., Yamanaka, S., Chambers, I., and Smith, A. (2009). Nanog is the gateway to the pluripotent ground state. Cell *138*, 722–737.

Sitzmann, J., Noben-Trauth, K., Kamano, H., and Klempnauer, K.H. (1996). Expression of B-Myb during mouse embryogenesis. Oncogene *12*, 1889–1894.

Sommer, C.A., Stadtfeld, M., Murphy, G.J., Hochedlinger, K., Kotton, D.N., and Mostoslavsky, G. (2009). Induced pluripotent stem cell generation using a single lentiviral stem cell cassette. Stem Cells *27*, 543–549.

Sommer, C.A., Sommer, A.G., Longmire, T.A., Christodoulou, C., Thomas, D.D., Gostissa, M., Alt, F.W., Murphy, G.J., Kotton, D.N., and Mostoslavsky, G. (2010). Excision of reprogramming transgenes improves the differentiation potential of iPS cells generated with a single excisable vector. Stem Cells *28*, 64–74.

Soufi, A., Donahue, G., and Zaret, K.S. (2012). Facilitators and impediments of the pluripotency reprogramming factors' initial engagement with the genome. Cell *151*, 994–1004.

Takahashi, K., and Yamanaka, S. (2006). Induction of pluripotent stem cells from mouse embryonic and adult fibroblast cultures by defined factors. Cell *126*, 663–676.

Tanaka, Y., Patestos, N.P., Maekawa, T., and Ishii, S. (1999). B-myb is required for inner cell mass formation at an early stage of development. J. Biol. Chem. *274*, 28067–28070.

Tarasov, K.V., Tarasova, Y.S., Tam, W.L., Riordon, D.R., Elliott, S.T., Kania, G., Li, J., Yamanaka, S., Crider, D.G., Testa, G., et al. (2008). B-MYB is essential for normal cell cycle progression and chromosomal stability of embryonic stem cells. PLoS One *3*, e2478.

Theunissen, T.W., van Oosten, A.L., Castelo-Branco, G., Hall, J., Smith, A., and Silva, J.C. (2011). Nanog overcomes reprogramming barriers and induces pluripotency in minimal conditions. Curr. Biol. *21*, 65–71.

Trapnell, C., Pachter, L., and Salzberg, S.L. (2009). TopHat: discovering splice junctions with RNA-Seq. Bioinformatics *25*, 1105–1111.

Unternaehrer, J.J., Zhao, R., Kim, K., Cesana, M., Powers, J.T., Ratanasirintrawoot, S., Onder, T., Shibue, T., Weinberg, R.A., and Daley, G.Q. (2014). The epithelial-mesenchymal transition factor SNAIL paradoxically enhances reprogramming. Stem Cell Reports *3*, 691–698.

Utikal, J., Polo, J.M., Stadtfeld, M., Maherali, N., Kulalert, W., Walsh, R.M., Khalil, A., Rheinwald, J.G., and Hochedlinger, K. (2009). Immortalization eliminates a roadblock during cellular reprogramming into iPS cells. Nature *460*, 1145–1148.

Vierbuchen, T., Ling, E., Cowley, C.J., Couch, C.H., Wang, X., Harmin, D.A., Roberts, C.W.M., and Greenberg, M.E. (2017). AP-1 transcription factors and the BAF complex mediate signal-dependent enhancer selection. Mol Cell *68*, 1067–1082.e12.

Viswanathan, S.R., Daley, G.Q., and Gregory, R.I. (2008). Selective blockade of microRNA processing by Lin28. Science *320*, 97–100.

Whyte, W.A., Orlando, D.A., Hnisz, D., Abraham, B.J., Lin, C.Y., Kagey, M.H., Rahl, P.B., Lee, T.I., and Young, R.A. (2013). Master transcription factors and mediator establish super-enhancers at key cell identity genes. Cell *153*, 307–319.

Zhang, Y., Liu, T., Meyer, C.A., Eeckhoute, J., Johnson, D.S., Bernstein, B.E., Nusbaum, C., Myers, R.M., Brown, M., Li, W., and Liu, X.S. (2008). Model-based analysis of ChIP-Seq (MACS). Genome Biol. *9*, R137.

STAR★METHODS

KEY RESOURCES TABLE

REAGENT or RESOURCE	SOURCE	IDENTIFIER
Antibodies		
anti- mouse CD44 APC	Thermofisher	Cat#17-0441-83; RRID:AB_469391
anti- mouse CD54 (ICAM-1)-biotin	Thermofisher	Cat# 13-0541-85; RRID:AB_466481
streptavidin-PE-Cy7	BD Bioscience	Cat# 557598; RRID:AB_10049577
Mouse IgG	Sta Cruz	Cat# sc-2025; RRID:AB_737182
mouse HA antibody	Abcam	Cat# Ab18181; RRID:AB_444303
Polyclonal B-Myb antibody	Sta Cruz	Cat# sc-724; RRID:AB_631985
Chemicals, Peptides, and Recombinant Proteins		
Dox hyclate	SIGMA	Cat# D9891
Ascorbic acid (Vitamin C)	SIGMA	Cat# PHR1008
LIF	Millipore	Cat# ESG1107
4-hydroxy Tamoxifen	SIGMA	Cat# H7907
GSK3 inhibitor	Millipore	Cat# CHIR99021
MEK 1 inhibitor	Millipore	Cat# PD0325901
AMPure XP beads	Beckman Coulter	Cat#A63882
Tagment DNA Buffer	Illumina	Cat#15027866
NEBNext High-Fidelity 2x PCR Master Mix	New England BioLabs	Cat#M0541S
Critical Commercial Assays		
Senescence B-galactosidase staining kit	Cell Signaling	Cat#9860
In situ Cell Death Detection Kit (TUNEL)	Roche	Cat#000000011684795910
Lenti-X qRT-PCR Titration Kit	Clontech	Cat# 631235
TruSeq Stranded mRNA with Ribo-Zero human/ mouse/rat assay	Illumina	Cat# 20020596
TruSeq RNA Sample Prep kit	Illumina	Cat#RS-122-2001
KAPA hyper Prep Kit	Kapa Biosystems	KK8500
KAPA Library Quantification kit	Kapa Biosystems	Cat#KK4824
MinElute Gel Extraction Kit	QIAGEN	Cat#28604
NextSeq500 High output 150 cycles	Illumina	Cat#FC-404-2002
NextSeq500 High output 75 cycles	Illumina	Cat#FC-404-2005
Deposited Data		
ATAC-seq data	This study	GEO: GSE107577
RNA-seq data	This study	GEO: GSE107578
Experimental Models: Cell Lines		
TNG MKOS mouse line	Keisuke Kaji lab	N/A
HEK293T human cell line	ATCC	Cat# CRL-3216, RRID:CVCL_0063
Experimental Models: Organisms/Strains		
mouse B6;129S4-*Pou5f1*$^{tm2(EGFP)Jae}$/J	The Jackson Laboratory	JAX:008214
Mouse B6: B-*myb*$^{+/\Delta}$	Paloma Garcia lab	N/A
Mouse B6: B-*myb*$^{+/F}$	Paloma Garcia lab	N/A
Mouse B6: B-*myb*$^{+/\Delta}$:*Oct4-GFP*^{Hm}	This study	N/A
Mouse B6: B-*myb*$^{+/F}$:*Oct4-GFP*^{Hm}	This study	N/A
Mouse: B6.129-*Gt(ROSA)26Sor*$^{tm1(cre/ERT2)Tyj}$/J	The Jackson Laboratory	JAX:008463
B-*myb*$^{+/\Delta}$:*Oct4-GFP*^{Hm}:*CreERT2*^{Hm}	This study	N/A

(*Continued on next page*)

REAGENT or RESOURCE	SOURCE	IDENTIFIER
Continued		
B-*myb*$^{+/F}$:*Oct4-GFP*^{Hm}:*CreERT2*^{Hm}	This study	N/A
Mouse B6: *P53*$^{+/-}$	Jorge Cammano lab	N/A
Oligonucleotides		
Sox2 5′ NdeI; GGT TTC TTA CAT ATG ATG TAT AAC ATG ATG GAG ACG GAG CTG AAG	This study	N/A
Sox2-E2A 3′; TTT CAA CAT CGC CAG CGA GTT TCA ACA AAG CGT AGT TAG TAC ATT GCC CAC TAC CCA TGT GCG ACA GGG GCA GTG TGC CGT TAA TGG CCG	This study	N/A
E2A-B-Myb 5′; CTT TGT TGA AAC TCG CTG GCG ATG TTG AAA GTA ACC CCG GTC CTA TGT CTC GGC GGA CGC GCT GCG AGG ATC TGG ATG	This study	N/A
B-Myb 3′ ClaI; GGT TTA TCG ATT CAG GAC AGA ATG AGG GTC CGA GAT G	This study	N/A
5′B-Myb; GGT TTG GAT CCA TGT CTC GGC GGA CGC GCT GCG AGG	This study	N/A
3′B-Myb2-P2A; CTC CTC CAC GTC TCC AGC CTG CTT CAG CAG GCT GAA GTT AGT AGC TCC GCT TCC GGA CAG AAT GAG GGT CCG AGA TGT GTG GCT G	This study	N/A
P2A-5′AmCyan; AGC CTG CTG AAG CAG GCT GGA GAC GTG GAG GAG AAC CCT GGA CCT ATG GCT CTT TCA AAC AAG TTT ATC GGA GAT	This study	N/A
TGFbr3-F; ATTTCCCCTGTGCCAATGTG	This study	N/A
TGFbr3-R; TGAGGGTGGGGAAGCATTTT	This study	N/A
COL11A1_F; AGGAGAGGAGAGAATGGG	This study	N/A
COL11A1_R; CGAGTGTGAAGCCCCTAAGA	This study	N/A
LEF1_1_F; CGGATTGGAGAACGAGGGT	This study	N/A
LEF1_1_R; TCAGTCTGTGGGCATCTTCA	This study	N/A
LEF1_2_F; CCCTTAGCAATTGTTTCACGGT	This study	N/A
LEF1_2_R; CCACTGGAGGTTGGCTGT	This study	N/A
COL6a3_F; CTATGAGCCCTGAAACCCCA	This study	N/A
COL6a3_R; CGGGTCTGTTTTGGGAAAGG	This study	N/A
GpIIb Neg_F; GATTCAGCCTTTCAGCAGCACTAC	This study	N/A
GpIIb Neg_R; AACTGTTTGTGGACGGAGTCACTG	This study	N/A
Pouf5F1(Oct4)	Applied biosystems	Mm00658129_gH
Klf4	Applied biosystems	Mm00516104_m1
Nanog	Applied biosystems	Mm02384862_g1
Myc	Applied biosystems	Mm00487804_m1
FGF4	Applied biosystems	Mm00438916_g1
Sall4	Applied biosystems	Mm01240680_m1
Cbx7	Applied biosystems	Mm00520006_m1
ETV5	Applied biosystems	Mm00465816_m1
Mybl2	Applied biosystems	Mm00485340_m1
lin28a	Applied biosystems	Mm00524077_m1
Thy1	Applied biosystems	Mm00493682_g1
Cdh1	Applied biosystems	Mm01247357_m1
Cdkn1a (P21)	Applied biosystems	Mm04205640_g1
Cdkn2a (P16)	Applied biosystems	Mm00494449_m1
Trp53 (P53)	Applied biosystems	Mm01731290_g1

(*Continued on next page*)

Continued

REAGENT or RESOURCE	SOURCE	IDENTIFIER
Col5a1	Applied biosystems	Mm00489342_m1
Col14a1	Applied biosystems	Mm00805269_m1
Postn	Applied biosystems	Mm01284919_m1
Fn1	Applied biosystems	Mm01256744_m1
Krt80	Applied biosystems	Mm04209123_m1
Krt8	Applied biosystems	Mm04209403_g1
Lef1	Applied biosystems	Mm00550265_m1
B2 microglobulin	Applied biosystems	Mm00437762_m1
Recombinant DNA		
pHAGE-2-EF1a-mSTEMCCA-loxP	George Murphy lab (Sommer et al., 2010)	http://www.bumc.bu.edu/kottonlab/vectors-and-plasmids/
pHAGE-2-EF1a-mSTEMCCA	George Murphy lab (Sommer et al., 2009)	http://www.bumc.bu.edu/kottonlab/vectors-and-plasmids/
pHAGE-2-EF1a-mOSK-Cherry-loxP	George Murphy lab (Sommer et al., 2010)	http://www.bumc.bu.edu/kottonlab/vectors-and-plasmids/
pHAGE-2-EF1a-ZsGreen	George Murphy lab	http://www.bumc.bu.edu/kottonlab/vectors-and-plasmids/
pRRL-PGK-IRES-Mybl2	This article	N/A
pHAGE-2-EF1a-mOSKB	This article	N/A
pHAGE-2-EF1a-AmCyan	This article	N/A
Fu-Tet-ON-Cre-P2A-ZsGreen	This article	N/A
Fu-Tet-ON	Addis et al., 2011	Addgene 43914
pHDM-tat1b	George Murphy lab	N/A
pREV	George Murphy lab	N/A
pHDM-VSV-G	George Murphy lab	N/A
pHDM-Hgpm2	George Murphy lab	N/A
Software and Algorithms		
Bowtie 2	Langmead and Salzberg, 2012	http://bowtie-bio.sourceforge.net/bowtie2/index.shtml
MACS2	Martin, 2011	https://github.com/taoliu/MACS
Homer	Heinz et al., 2010	http://homer.ucsd.edu/homer/motif/
Tophat v2.0.10	Trapnell et al., 2009	http://ccb.jhu.edu/software/tophat
Cufflinks v2.2.1	Trapnell et al., 2009	http://cole-trapnell-lab.github.io/cufflinks/announcements/protocol-paper/
Cuffdiff	Trapnell et al., 2009	http://cole-trapnell-lab.github.io/cufflinks/manual/
Bedtools	Quinlan and Hall, 2010	http://bedtools.readthedocs.io/en/latest/
Flowjo	FLOWJO LLC	https://www.flowjo.com/
Illustrator	Adobe System Software Ireland	http://www.adobe.com/cn/products/cs6/illustrator.html
ZEN	Zeiss	https://www.zeiss.com/microscopy/int/software-cameras.html
GraphPad Prism 6.0	GraphPad Software	https://www.graphpad.com/scientificsoftware/prism/
See also RNA-seq and data analysis section	This study	N/A
See also ATAC-seq and data analysis section	This study	N/A
Other		
Published reprogramming ATAC-Seq and ChIP-Seq data	Knaupp et al., 2017	GSE101905
Published reprogramming ATAC-Seq	Chronis et al., 2017	GSE90892

(*Continued on next page*)

Continued

REAGENT or RESOURCE	SOURCE	IDENTIFIER
Published mESC ChIP-Seq	Whyte et al., 2013	GSE44286
Published reprogramming AP-1 ChIP-Seq data	Liu et al., 2015	GSE50776
Published MEF ATAC-Seq and AP-1 ChIP-Seq data	Vierbuchen et al., 2017	GSE83295

CONTACT FOR REAGENTS AND RESOURCE SHARING

Further information and requests for reagents should be directed to and will be fulfilled by Lead contact, Paloma Garcia, at p.garcia@bham.ac.uk.

EXPERIMENTAL MODEL AND SUBJECT DETAILS

Cell lines and primary cells

293T cells were grown in Dulbecco's modified eagle medium (DMEM) with 1% Penicillin/Streptomycin (5000U/ml), 2mM L-glutamine (all from GIBCO) and 10% fetal bovine serum (FBS, Sigma). Referred to herein as 293T medium.

MEFs were obtained from embryos at day E12.5 and grown in DMEM with 1% Penicillin/Streptomycin (5000U/ml), 2mM L-glutamine, 10%–20% FBS and 0.1mM 2-mercaptoethanol (Sigma). MEFs were used prior passage 2 for all reprogramming studies.

MEFs undergoing somatic reprogramming were grown in iPSC medium: DMEM with 1% Penicillin/Streptomycin (5000U/ml), 2mM L-glutamine, 5% ESC qualified FBS (Hyclone, Fisher Scientific), 1% non-essential amino acids (GIBCO), 0.1mM 2-mercaptoethanol and 0.01% LIF (Leukemia inhibitory factor, 10^6U/ml, Millipore).

Mice used for the generation of MEFs

Oct4-GFP mice (B6;129S4-*Pou5f1*$^{tm2(EGFP)Jae}$/J, The Jackson Laboratory) were crossed with B-*myb*$^{+/\Delta}$ and B-*myb*$^{+/F}$ mice (García et al., 2005), for two rounds to generate B-*myb*$^{+/\Delta}$:*Oct4-GFP*^{Hm} and B-*myb*$^{+/F}$:*Oct4-GFP*^{Hm} mice. Next these mice were crossed with CreERT2 mice (B6.129-*Gt(ROSA)26Sor*$^{tm1(cre/ERT2)Tyj}$/J, The Jackson Laboratory) to generate B-*myb*$^{+/\Delta}$:*Oct4-GFP*^{Hm}:*CreERT2*^{Hm} and B-*myb*$^{+/F}$:*Oct4-GFP*^{Hm}:*CreERT2*^{Hm} mice. These were then used to set up timed matings to obtain embryos at E.12.5 with B-*myb*$^{F/\Delta}$:*Oct4-GFP*^{Hm}:*CreERT2*^{Ht} or B-*myb*$^{+/\Delta}$:*Oct4-GFP*^{Hm}:*CreERT2*^{Ht} to be used for experiments. *P53*$^{+/-}$ mice were a generous gift from Jorge Caamano, University of Birmingham. *P53*$^{+/-}$ mice were crossed together to generate embryos that were *P53*$^{-/-}$. Mice were maintained on a C57/BL6 background and genotyped by Transnetyx. All animals were maintained under an animal project license in accordance with UK legislation.

METHOD DETAILS

Lentiviral production

The Oct4,-Sox2-Klf4-c-Myc (OSKM), Zs-Green (GFP) and Oct4-Sox2-Klf4-mCherry (OSK) lentiviral backbone plasmids and the plasmids used for packaging the lentiviruses can be found in Table S1. The lentivirus particles used in this study were grown as previously described (Mostoslavsky et al., 2006). Virus with fluorescent reporter genes were titrated by flow cytometry using a Beckman Coulter Cyan or BD biosciences LSRFortessa flow cytometer. Viruses without fluorescent reporter genes were titrated using a Lenti-X qRT-PCR Titration Kit (Clontech).

Primary reprogramming

MEFs were reprogrammed using lentiviral particles containing one of four backbones (pHAGE-2-E1a-mOSKM-loxP, pHAGE-2-E1a-mOSKM, pHAGE-2-E1a-mOSKM-Cherry-loxP or pHAGE-2-E1a-mOSKB). Reprogramming was carried out in 0.1% gelatin coated 6 well or 12 well plates with 10^5 or 5×10^4 MEFs per well, respectively. MEFs were transduced in the presence of 5 μg/ml polybrene (Sigma) with 2 and 20 lentiviral particles per cell diluted in full MEF medium one day after plating. The cells were incubated at 37°C for 24 hours before the infections were stopped by aspiration of the lentivirus containing medium and addition of fresh iPSC medium. The medium on the cells was changed every 48 hours. If colonies were to be isolated the medium was changed to ESC medium when colonies were observed using an inverted microscope (Zeiss, Axiovert 25).

Somatic reprogramming using Tg-reprogrammable MEFs

10% MEFs containing the four reprogramming factors under dox control (Chantzoura et al., 2015) were mixed with 90% wild-type MEFs and plated onto 0.1% gelatin coated six well plates at a cell density of 10^5 per well. MEFs were infected with pHAGE-2-E1a-mybL2-Linker-HA-EF1a-Amcyan or pHAGE-2-E1a-Amcyan control virus twenty-four hours prior the addition of dox. Reprogramming was initiated by addition of 300ng/ml dox hyclate (Sigma) to the culture medium. The cells were cultured in iPSC medium

with 10 μg/ml vitamin C (Sigma). The cells were given fresh medium with dox every 48 hours and reprogramming progress was monitored by inverted fluorescence microscopy and analyzed by flow cytometry.

Flow cytometric analysis of reprogramming process

MEFs undergoing reprogramming were stained with anti-CD44-APC (Clone IM7) and anti-CD54-biotin (clone YN1/1.7.4) antibodies (both from Thermofisher) diluted in 5% FBS/PBS for 15 minutes on ice. After washing cells were resuspended and incubated with anti-streptavidin-PE-Cy7 antibody (BD Boscience) diluted in 5% FBS/PBS for 10 minutes. After three washes cells were resuspended in 400 μL of 5% FBS in PBS and filtered through a 50 μL Celltrics filter (Partec). The CD44 and CD54 expression was analyzed by BD biosciences LSRFortessa flow cytometer.

Generation of pHAGE-2-EF1a-mOSKB, pHAGE-2-EF1a-AmCyan, pHAGE-2-EF1a-Mybl2-HA-P2A-EF1a-AmCyan

PCR was used to clone *Sox2*, *Mybl2*, and *AmCyan* from plasmid DNA with the following conditions: 30 cycles of (98°C for 10 s, 60°C for 15 s, 68°C for 3 minutes). Each reaction contained 10ng of plasmid DNA (pHAGE-2-E1a-mOSKM-Cherry-loxP, pMB21, pAmCyan, respectively), 4 μL dNTPs (2.5mM each, Takara Bio Inc), 1 μL GXL polymerase (1.25U, Takara Bio Inc), 10 μL PrimeSTAR GXL Buffer (1X, Takara Bio Inc), 0.1 μL forward primer, 0.1 μL reverse primer (key resources table) and dH_2O up to 50 μL final volume.

PCR was used to combine the modified *Sox2* and M*ybl2* fragments, the modified *AmCyan* and B-*myb* fragments with the following conditions: 30 cycles of (98°C for 10 s, 60°C for 15 s, 72°C for 4 minutes). The reaction contained 10ng of each purified fragment, 4 μL dNTPs (2.5mM each, Takara Bio Inc), 1 μL GXL polymerase (1.25U, Takara Bio Inc), 10 μL PrimeSTAR GXL Buffer (1X, Takara Bio Inc), 0.1 μL Sox2 5′-NdeI primer, 0.1 μL b-myb 3′ ClaI primer and dH_2O up to 50 μL final volume.

PCR was also used to clone a gateway cassette into the pHAGE-2-EF1a-ZsGreen-W lentiviral backbone in place of the ZsGreen gene with the following conditions: 35 cycles of (98°C for 10 s, 55°C for 5 s, 72°C for 9 s). Each reaction contained 200ng plasmid DNA (pMX-GW), 25 μL PrimeStar Max polymerase premix (2X, Takara Bio Inc), 0.1 μL forward primer. 0.1 μL reverse primer and dH_2O up to 50 μL final volume.

Cloning products were separated by gel electrophoresis on 1% agarose gels, excised from the geland purified using a QIAGEN Gel extraction Kit.

Generation of Fu-TetO-Cre-P2A-ZsGreen

PCR was used to clone *NLS-Cre*, *P2A*, and *ZsGreen* from plasmid DNA with the following conditions: 35 cycles of (98°C for 10 s, 60°C for 15 s, 72°C for 15 minutes). Each reaction contained 100ng of plasmid DNA (pHAGE-2-E1a-mOSKM-Cherry-loxP, pMB21, pAmCyan, respectively), 25 μL Polymerase Master Mix (PrimeStar, Takara), 0.2 μM forward primer, 0.2 μM reverse primer (key resources table) and dH_2O up to 50 μL final volume.

PCR was used to combine the *NLS-Cre*, *P2A*, and *ZsGreen* fragments with the following conditions: 30 cycles of (98°C for 10 s, 59°C for 15 s, 72°C for 4 minutes). The reaction contained 50ng of each purified fragment, 25 μL Polymerase Master Mix (PrimeStar, Takara), 0.2 μM forward primer, 0.2 μM reverse primer containing 15bp overlapping the pENTRY2B2 vector (key resources table) and dH_2O up to 50 μL final volume.

Gibson Assembly

Gibson assembly was used to combine the gateway cassette fragment cloned from the pMX-GW plasmid into pHAGE-2-EF1a plasmid linearized with NcoI and BamHI restriction enzymes. Gibson assembly reaction contents: 150ng insert (GW cassette), 50ng linearized vector (pHAGE-2-EF1a plasmid cut with NcoI and BamHI), 10 μL Gibson master mix (2X, NEB) and dH_2O up to 20 μL final volume. The reaction was incubated at 50°C for 1 hour. The reaction mixture was diluted 1:4 with dH_20 and then 2 μL was used to transform OneShot Ccdb resistant competent bacteria (Invitrogen). Gibson assembly was also used to subclone the NLS-Cre-P2A-ZsGreen fragment into the pENTRY vector (linearized with BamHI and NotI).

Gateway cloning

Gateway cloning was used to move genes of interest from *attL* entry vectors to the generated *attR* destination vector, pHAGE-2-EF1a-GW or Fu-TetOn (Addis et al., 2011) vector. 150ng of entry vector and 150ng of destination vector were diluted with TE buffer to a final volume of 8 μl. 2 μL of LR Clonase II enzyme mix (5x, Invitrogen) was added to the sample. The DNA and enzyme mixture was incubated for 1 hour at 25°C. 1 μL of Proteinase K (Invitrogen) was added and the sample was mixed and then incubated at 37°C for 10 minutes. 1 μL of the reaction was transformed into competent bacteria. Any DNA that was cloned by PCR was sequenced to confirm lack of mutations.

Cell Sorting

Cells were sorted after viral transduction or transfection by FACS on a MoFlo XDP high speed cell sorter (Beckman Coulter). Cells were sorted for positive expression of the fluorescent reporter protein after gating for live cells and singlets.

Quantitative RT-PCR determination of mRNA levels

Real time PCR was carried out using TaqMan PCR reaction buffer (Applied Biosystems). The reaction was carried out in a Stratagene Mx3000P machine. At least two separate reactions with three replicates per sample were run. Relative gene expression was calculated as $2^{-\Delta\Delta Ct}$ values with β2 microglobulin as a control. Primer sequences can be obtained from the key resources table.

AP staining

AP staining was performed according to standard protocols. In brief, medium was aspirated from the well before washing with cold PBS. The cells were fixed in 500μl cold neutral formalin buffer (0.1M Na_2HPO_4, 25mM $Na_2HPO_4.H_2O$, 4% Paraformaldehyde) for 15 minutes. The fixative was removed and the cells were rinsed once with cold dH_2O, and then left in dH_2O for 15 minutes. The dH_2O was removed and 500μl of staining solution (0.1mM Naphthol AS-MX phosphate, 27.3mM N,N-Dimethylformamide (DMF), 0.7mM Fast Red Violet LB Salt, 0.1M Tris-HCl pH 8.3) was added to the cells and then incubated at room temperature for 30 minutes. The staining solution was removed and the cells were washed once with dH_2O and then allowed to air dry. Red positively stained colonies were counted to assess the efficiency of reprogramming.

Senescence staining

Reprogrammed MEFs were stained for senescence by using a Senescence β-Galactosidase Staining Kit (Cell Signaling Technology) according to the manufacturer's instructions. The stained cells were imaged using an inverted bright field microscope at 20x magnification with an attached camera.

Apoptosis staining (TUNEL)

An *In Situ* Cell Death Detection kit (TUNEL, Roche) was used to assess whether the viral transduction of MEFs was causing apoptosis. MEFs induced to reprogram were grown on 0.1% gelatin coated 10mm coverslips in 4 well plates were stained according to the manufacturer's instructions. The stained cells were counterstained using Prolong antifade reagent with DAPI (Life Technologies). Samples were analyzed on a Zeiss LSM 510 Meta confocal microscope.

ATAC-seq

To profile open chromatin, we modified the previously published ATAC-seq protocol (Buenrostro et al., 2015): Tg-reprogrammable MEF (three independent biological replicates) were infected with lentivirus containing Mybl2HA-Amcyan or AmCyan genes. One day later starting of reprogramming was initiated by addition of dox and 3 days after reprogramming 50,000 cells were sorted based on AmCyan and mOrange fluorescent proteins and nuclei were isolated as follows: 50,000 cells were pelleted by centrifuging at 500 × g and 4°C for 10 min. Cells were then washed with 50 μL of cold PBS before being pelleted again as outlined above. Pellets were then resuspended in 500 μL of Lysis buffer (10mM TrisHcl ph7.4, 10mM NaCl, 3mM MgCl2, 0.1% IGEPAL) and placed on ice for 10 min. Finally, cells were pelleted by centrifugation at 500 × g and 4°C for 10 min before the supernatant was discarded. The isolated nuclei were then resuspended in a 50 μL reaction buffer containing 5 μL of Tn5 transposase (Illumina), 2% Digitonin (Promega) and 25 μL of TD buffer (Nextera sample preparation kit from Illumina) and incubated at 37°C for 30 minutes. The DNA was then purified using DNA QIAGEN MiniElute kit and eluted into 10 μL of elution buffer. For library amplification, 10 μL of DNA was combined with 2.5 μL of indexing primers (Nextera Customized PCR Primer 1 and barcode Nextera PCR Primer 2), 25 μL of NEBNextHigh-Fidelity 2x PCR Master Mix (New England Biolabs). DNA was then amplified for 8 cycles to enrich the tagmented DNA fragments. A PCR clean-up was then performed using AMPure XP beads (Beckman Coulter), and the small fragments were then resuspended in 32.5 μL of resuspension buffer (provided in the Nextera kit). DNA was quantified using a Qubit fluorometer (Life Technologies), and library sizes were then determined using TapeStation (Agilent Technologies). Sequencing was performed using a NextSeq 500 to obtain an average of 40 million reads per sample.

Alignment of ATAC-seq data

Cutadapt (Martin, 2011) was used in paired end mode to trim the adaptor sequence and separate sequences where both read ends were retained from sequences. Reads that aligned to the mitochondrial genome were removed in Bowtie 2 (release 2.2.5)(Langmead and Salzberg, 2012). Bowtie 2 was then used first to align the trimmed paired-end data and then the single-ended read data to the mm9 reference genome. ATAC-seq peaks were identified using MACS2 (Zhang et al., 2008).

Peak detection and filtering; coverage track generation

MACS v2.1.0 was used to call peaks using optional arguments:–keep-dup = auto -B–trackline -f BAMPE -g mm. bedtools was used to filter ENCODE blacklisted sequencing regions. Bedtools and MACS bdgdiff were used to compare peak differences.

Two-way fold change analysis

Two-way fold change analyses for AmCyan and Mybl2 samples were performed as previously described for ATAC-Seq (Brignall et al., 2017), using all peaks originating from MACS2.

Motif discovery and motif density plots

Motif discovery was performed with the findMotifsGenome (Heinz et al., 2010) on regions top 10% or bottom 10% fold change versus control, using default parameters. For density plots from all peaks or 10 top 10% or bottom 10% fold change versus control, the annotatePeaks function of Homer was used with the -size 200 -hist 10 -m < motif > options and motif densities were plotted in excel.

RNA-seq

Tg- reprogrammable MEF were infected with lentivirus containing Mybl2HA-Amcyan or AmCyan genes. One day later starting of reprogramming was initiated by addition of dox and 3 days after reprogramming cells were sorted based on AmCyan and mOrange fluorescent proteins. RNA from two independent biological replicates was extracted from cells using Triazol and cleaned using RNeasy MinElute CleanUp kit by QIAGEN. The RNA was then quantified using a Agilent Technologies 2100 Bioanalyzer. 1 ug of total RNA was used for the RNA-seq. RNA-seq was then carried out using the TruSeq Stranded mRNA with Ribo-Zero human/mouse/rat assay (Illumina) following the manufacturers protocol. Samples were then paired-end sequenced using an Illumina HiSeq 2500 instrument (100 plus 100 cycles, plus indices). We sequenced an average of 22 million reads per library and aligned them to the mouse reference genome (mm10) using TopHat v2.0.10 using options: -r 100–library-type = fr-firststrand. Next, Cufflinks v2.2.1 was used to assemble transcripts using options:–library-type = fr-firststrand. The assembled transcripts from each sample were merged using Cuffmerge v2.2.1. Finally, Cuffdiff v2.2.1 was used to quantify differential gene expression using options:–library-type = fr-firststrand. The complete table of differential gene expression is available at GSE107577.

Differential gene expression scatterplot was generated using metaseq 0.5.5.4 python package. Significant gene heatmap was generated using log2 (FPKM +1) data per replicate. The heatmap was plotted using seaborn.clustermap python package.

Gene set enrichment analysis

RNA-seq expression was ranked based on fold change between control and treated samples. This ranked list was used to perform GSEA (v3.0) using the hallmark gene set from the Molecular Signatures Database v6.1.

Chromatin Immunoprecipitation

Chromatin immunoprecipitation were performed as previously described (Lorvellac et al., 2010). In this procedure, immortalized MEFs infected with a Mybl2HA-Amcyan lentivirus were collected 48h post-infection for chromatin preparation using double cross-linking. Cells were first washed with PBS and then cross-linked with Di(N-succinimidyl) glutarate (DSG) (Sigma, 8424-500MG-F). For each assay, 2 x10^7 cells were suspended in 30mls PBS and incubated with 250 μL DSG (50mg/500 μlDMSO) on a rotating wheel for 45 minutes at room temperature. After this step cells were washed 4 times with PBS and then resuspended in 10mls of PBS and used for the second cross-linking with 1% Formaldehyde for 10 minutes at room temperature. Cross-linking was then terminated by adding 4 volumes of cold PBS+0.125 M Glycine. Following this step cells were resuspended in 300 μL of ice-cold ChIP Lysis Buffer (50mM HEPES-KOH pH 7.5, 1mM EDTA pH 8, 1% Triton X-100, 0.1% Na Deoxycholate and proteinase inhibitor cocktail (Roche UK, Burgess Hill, UK)), and sonicated at 4°C using a Bioruptor (Diagenode, Liege, Belgium) to generate fragments an average length of 400-500 bp (10 min with 30 s "ON" and "OFF" cycles, power setting high). For the immunoprecipitation, 25 μg of chromatin were diluted in 5 volumes of ChIP Dilution Buffer (1% Triton X-100, 2mM EDTA pH 8, 150mM NaCl, 20mM Tris-HCl pH8 and proteinase inhibitor cocktail) plus 15 μL of Protein A Agarose and 15 μL of Protein G Agarose and incubated for 1 hour at 4°C with rotation. After this, the mix was centrifuged for 1 min at 3500rpm at 4°C and the supernatant was collected and incubated with 8 μg of Mouse IgG (sc-2025) for 2 hours at 4°C with rotation. At the end of this incubation, 15 μL of Protein A Agarose and 15 μL of Protein G Agarose were added to the mix followed by 1 hour incubation at 4°C with rotation, after which the mix was centrifuged and the supernatant collected. During this procedure, 8 μg of mouse HA antibody (Abcam, Ab18181) was incubated in 400 μL of ChIP Dilution Buffer supplemented with 10 μL of Protein A Agarose and 10 μL of Protein G Agarose and incubated for 4 hours at 4°C with rotation. The anibody complexes where then washed three times with ice-cold Dilution Buffer. At this stage, 10% of the material was separated for the INPUT control and the remaining precleared chromatin and the antibody complexes were combined and incubated overnight at 4°C with rotation. The day after the IPs were subjected to washes with ChIP Wash Buffers (0.1% SDS, 1% Triton X-100, 2mM EDTA, 20mM Tris-HCl pH 8, 150 mM NaCl) containing increasing NaCl concentrations, these being 150mM, 500mM, 600mM and 750mM, followed by a final wash with 10mM Tris-HCl pH 8, 10mM EDTA. After this step, IPs were resuspended in Q2 Elution Buffer (10mM Tris-HCl pH 7.5, 5mM EDTA, 5mM Na-Butirate, 50mM NaCl, 1% SDS and RNase A 1 μg/ml final) and incubated at 68°C on thermomixer at 1300rpm. At the end of this incubation, 1 μL of Proteinase K (15mg/ml, Roche) was added and IPs were further incubated for 2 hours at 68°C on thermomixer at 1300rpm. After this step, IP DNA was purified using a standard Phenol/Cloroform extraction followed by Ethanol precipitation. In the case of ChIP-seq assays, IP material was subjected to library preparation using KAPA hyper Prep Kit and run on an Illumina Hiseq 2500 sequencer.

QUANTIFICATION AND STATISTICAL ANALYSIS

All data shown are presented as mean ± SEM, all the statistical details of experiments can be found in the figure legends. When comparing datasets, two-tailed unpaired Student's t test was applied using GraphPad Prism software, unless indicated.

No statistical method was used to estimate the sample size. No specific randomization or blinding protocol was used. N indicates the numbers of independent biological replicas per experiment unless otherwise indicated. $p \leq 0.05$ was considered statistically significant. Significance tests were performed on all samples and therefore graphs lacking p values indicate results were not statistically significant.

DATA AND SOFTWARE AVAILABILITY

The accession number for the RNA-seq, and ATAC-seq data reported in this paper is GEO: GSE107577.

Cell Reports, Volume *24*

Supplemental Information

Fine-Tuning Mybl2 Is Required for Proper Mesenchymal-to-Epithelial Transition during Somatic Reprogramming

Carl Ward, Giacomo Volpe, Pierre Cauchy, Anetta Ptasinska, Ruba Almaghrabi, Daniel Blakemore, Monica Nafria, Doris Kestner, Jon Frampton, George Murphy, Yosef Buganim, Keisuke Kaji, and Paloma García

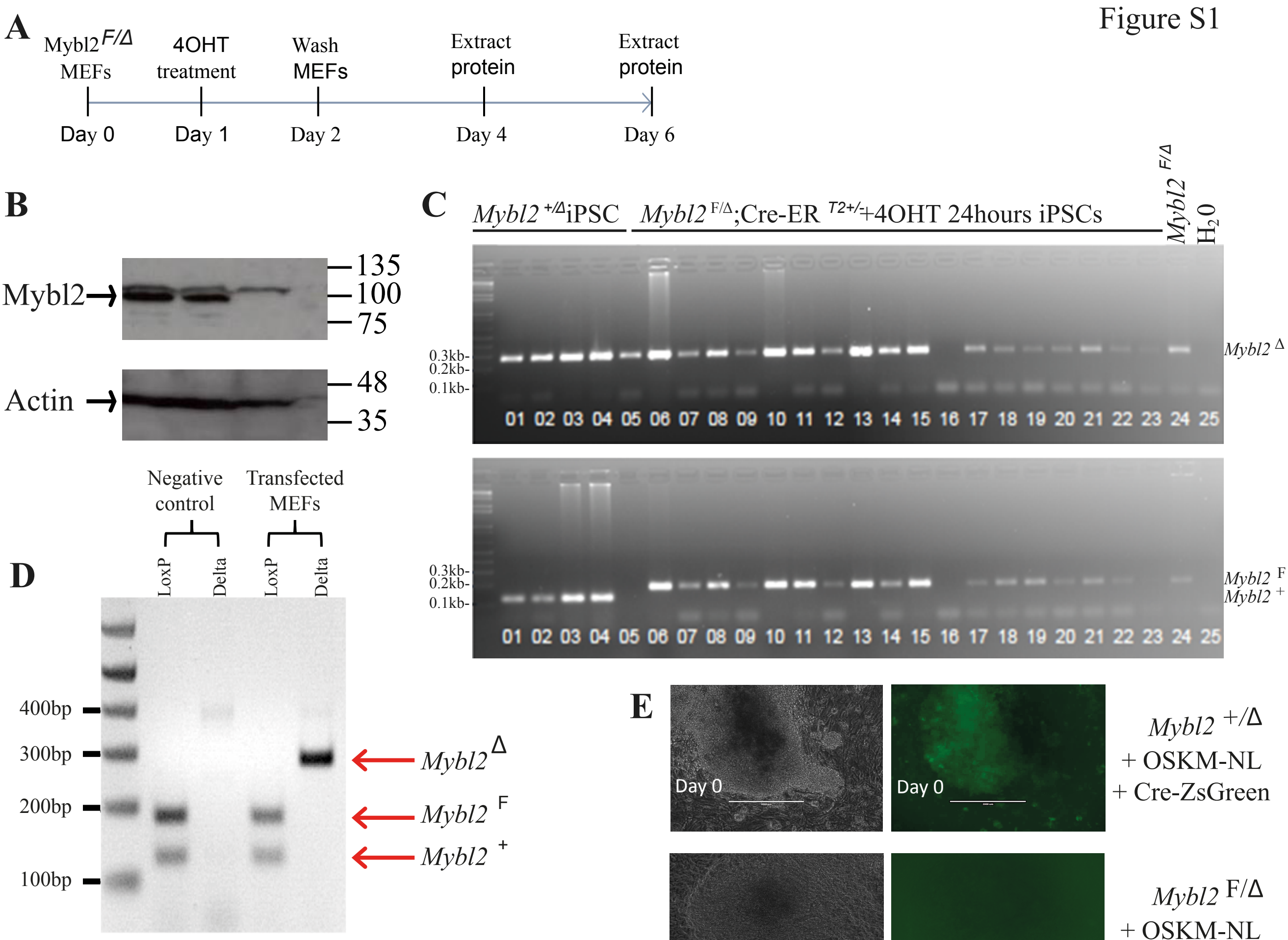

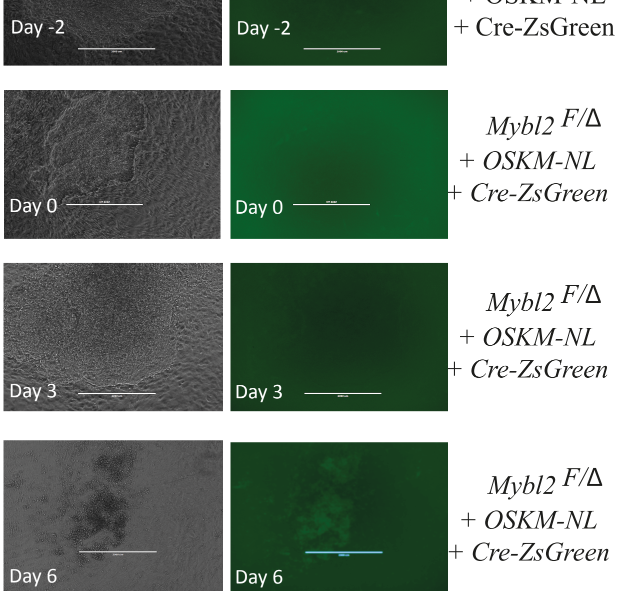

Figure S1. Mybl2 is required for reprogramming, Related to Figure 1. (A) Schematic representation of the strategy. (B) *Mybl2*$^{F/\Delta}$ Cre-ERT2$^{+/-}$ MEFs untreated (Lane 1), *Mybl2*$^{F/\Delta}$ Cre-ERT2$^{+/-}$ MEFs treated with 500nM 4-hydroxytamoxifen (4OHT) for 24h. 48 hours (Lane 2) or 96 hours (Lane 3) after 4OHT treatment. Western blotting with antibodies against Mybl2 or Actin. (C) PCR to detect the deleted *Mybl2*$^{\Delta}$ allele, one fragment at 240bp shows that there is a *Mybl2*$^{\Delta}$ allele present (upper) and PCR to detect the *Mybl2*^{WT} allele and *Mybl2*^{F} alleles, one fragment at 143bp shows that there is a *Mybl2*^{F} allele present (lower panel). (D) Validation of Fu-TetO-CreZsGreen construct. *Mybl2*$^{F/+}$ MEFs were transfected with the Fu-TetO-CreZsGreen or control vector. PCR for detection of the *Mybl2*$^{\Delta}$, *Mybl2*^{F} and *Mybl2*$^{+}$ alleles was performed. (E) Representative images of iPSC colonies from *Mybl2*$^{F/\Delta}$ and *Mybl2*$^{+/\Delta}$ transduced with dox inducible CreZsGreen lentiviral particles (MOI 10) together with OSKM lacking loxP sites (OSKM-NL) (MOI 2).

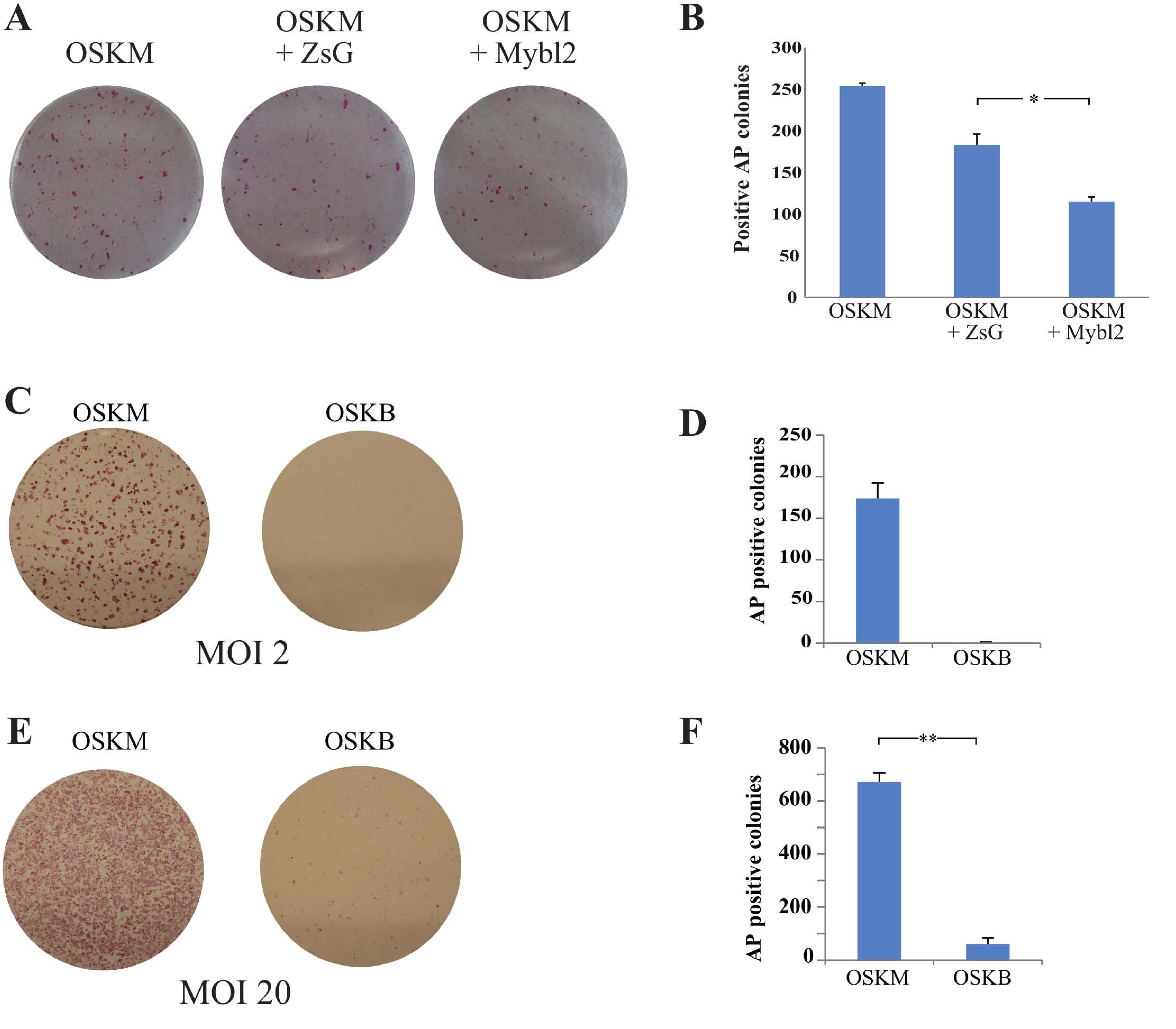

Figure S2. Alkaline phosphatase staining of reprogrammed wt and *p53* $^{-/-}$ MEFs, Related to Figure 2.
(A) MEFs were transduced with OSKM (left panel), OSKM + Zs-Green (middle panel) or OSKM + Mybl2 (right panel) lentiviralparticles (all MOI 1), then tested for pluripotency by alkaline phosphatase staining at day 10. Plates showing positive AP staining (red). (B) Graph representing the positive colony counts. (C) *p53* $^{-/-}$MEFs were transduced with OSKM, OSK or OSKB lentiviral particles at MOI 2 (C-D) or MOI 20 (E-F) and allowed to reprogram for 14 days before staining for AP activity. Quantification of alkaline positive colonies at MOI 2 (D) or MOI 20 (F) are shown on the histograms. N=3 biological replicates. Error bars represent SEM. *<0.05 by unpaired two-tailed T-test.

Figure S3

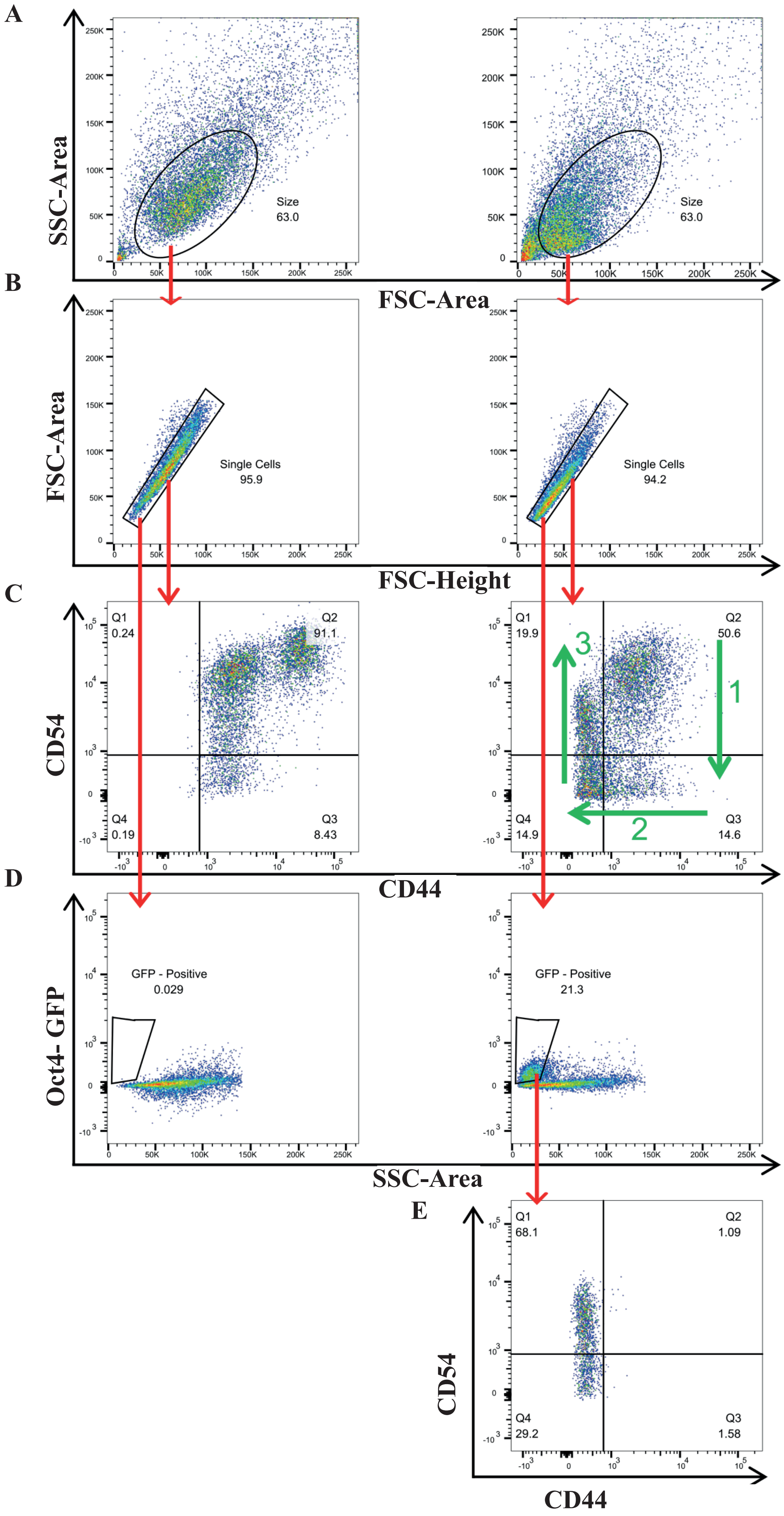

Figure S3. Gating strategy used in flow cytometry for reprogramming kinetics studies, Related to Figure 3.
Cells were gated based on their size and granularity (A, Live), then for doublet discrimination (B), then shown as dot plots representing their expression of CD44 and CD54. Green arrows show progression od CD44/CD54 expression during reprogramming (C). Reprogramming MEFs were gated for GFP expression driven by endogenous Oct4 promoter (D, GFP positive population). (E) Dot plot showing CD44/CD54 expression in GFP positive pre-iPSCs and iPSCs.

Figure S4

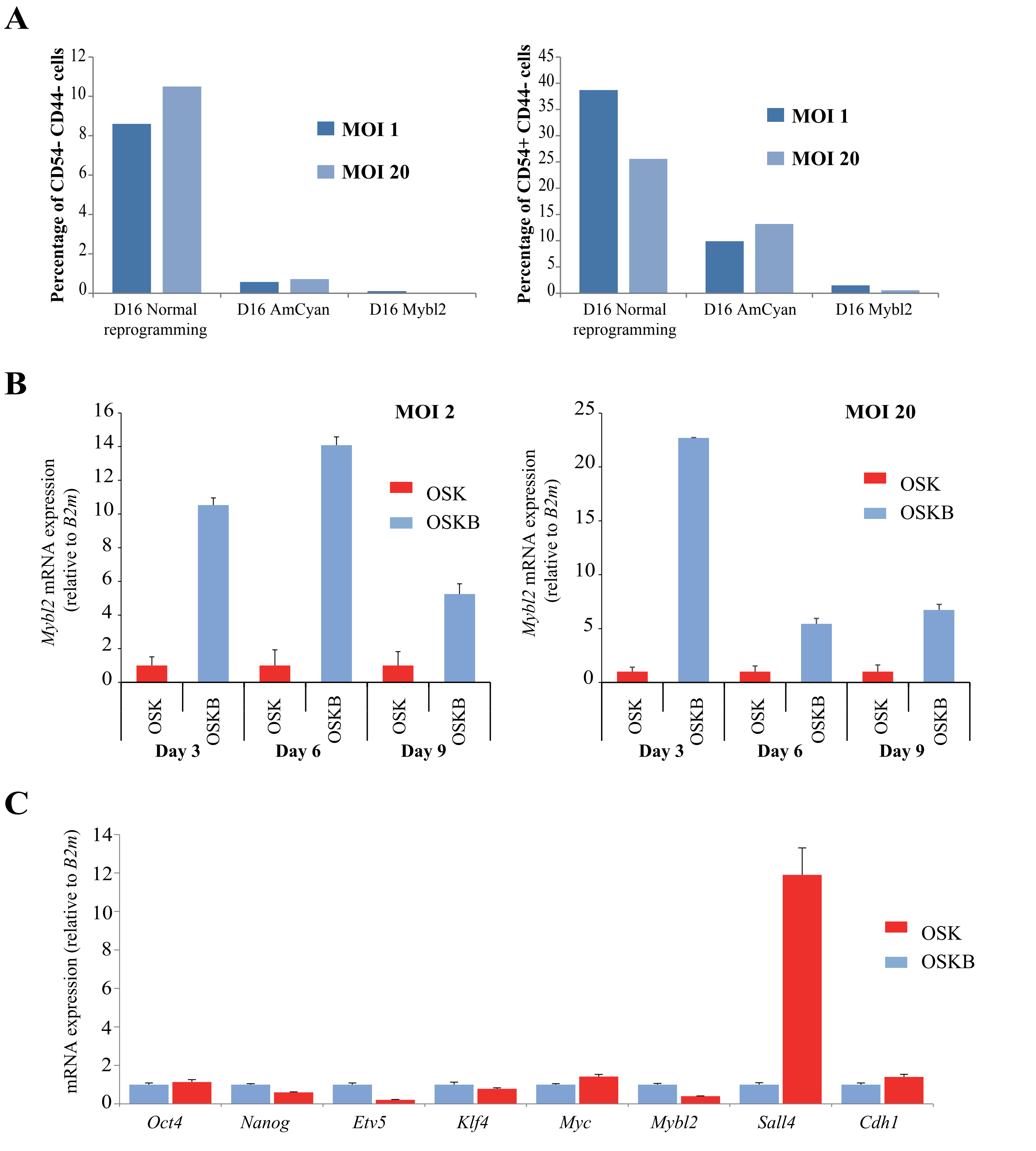

Figure S4. Somatic reprogramming is impaired when Mybl2 is overexpressed, Related to Figure 3.
(A) Somatic reprogramming is impaired using different Mybl2 expression levels but same OSK levels. MEFs were infected with OSK (harbouring Cherry reporter gene) at MOI 1 and Mybl2-AmCyan or AmCyan control lentiviral particles at MOI 1 and MOI 20. 16 days later, CD44/CD54 expression was assessed by flow cytometry. Histograms show the percentage of CD44-/CD54- (pre-iPSCs) and CD44-/CD54+ (iPSCs). (B) Real time PCR showing fold change in *Mybl2* expression levels during somatic reprogramming in OSKB in comparison to OSK sample (data from main Figure 3). Results show gene expression realtive to ESCs expression and normalized to the house keeping gene *β2-microglobulin*. (C) RNA expression of pluripotent genes in an individual iPSC clone derived from OSKB MOI 20 after excision of the lentiviral cassette measured using qRT-PCR.Values are relative to an ESC line and normalized against the *β2-microglobulin* gene. N=3 technical replicates.

Figure S5

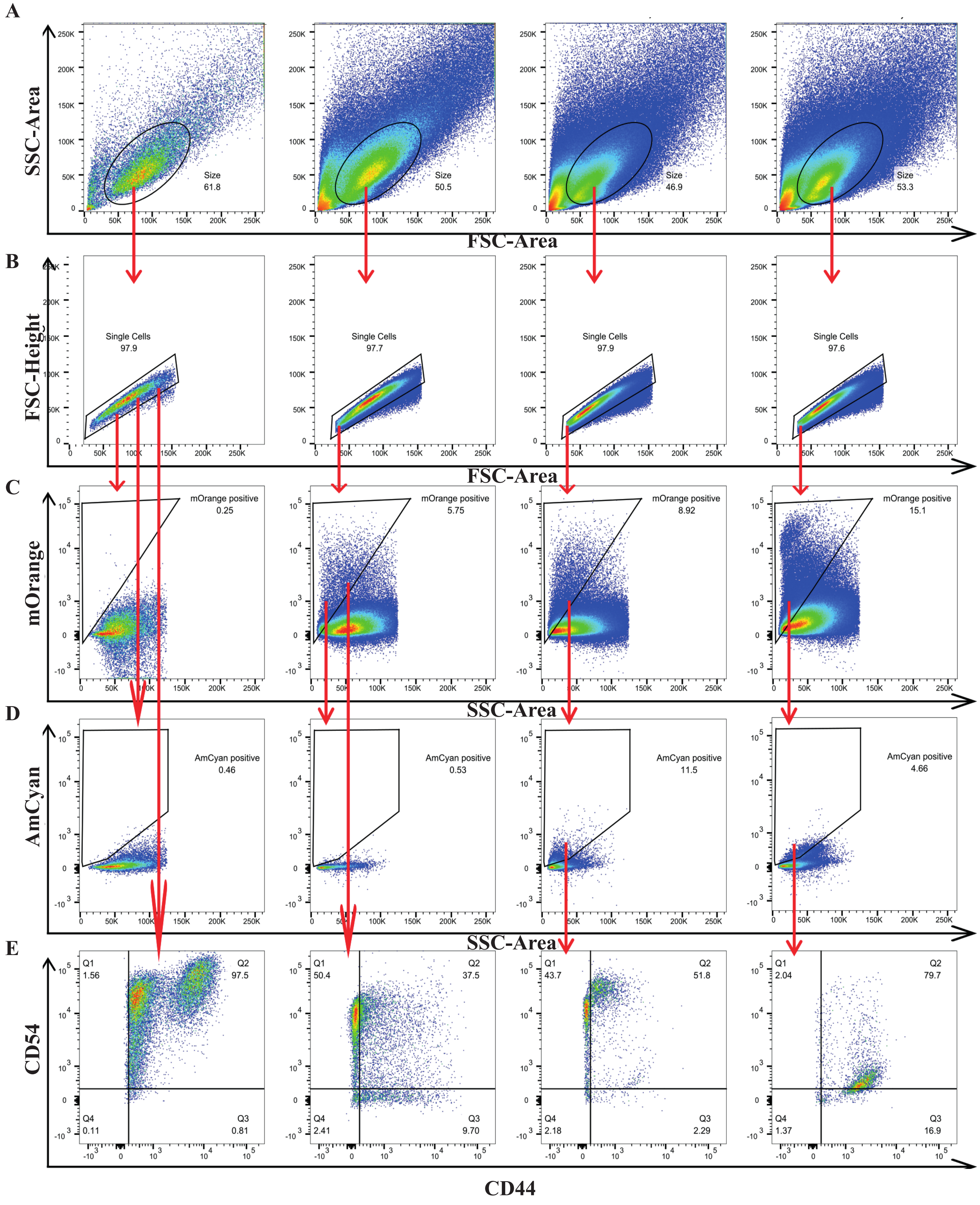

Figure S5. Gating strategy with Tg-reprogrammable MEFs (MKOS), Related to Figure 4.
Cells were gated based on size and granularity to exclude debris (A) and gated to identify single cells (B). Cells were gated for mOrange expression (C), then AmCyan expression (D) and finally shown as a dot plot based on their CD44 and CD54 expression (E).

Figure S6

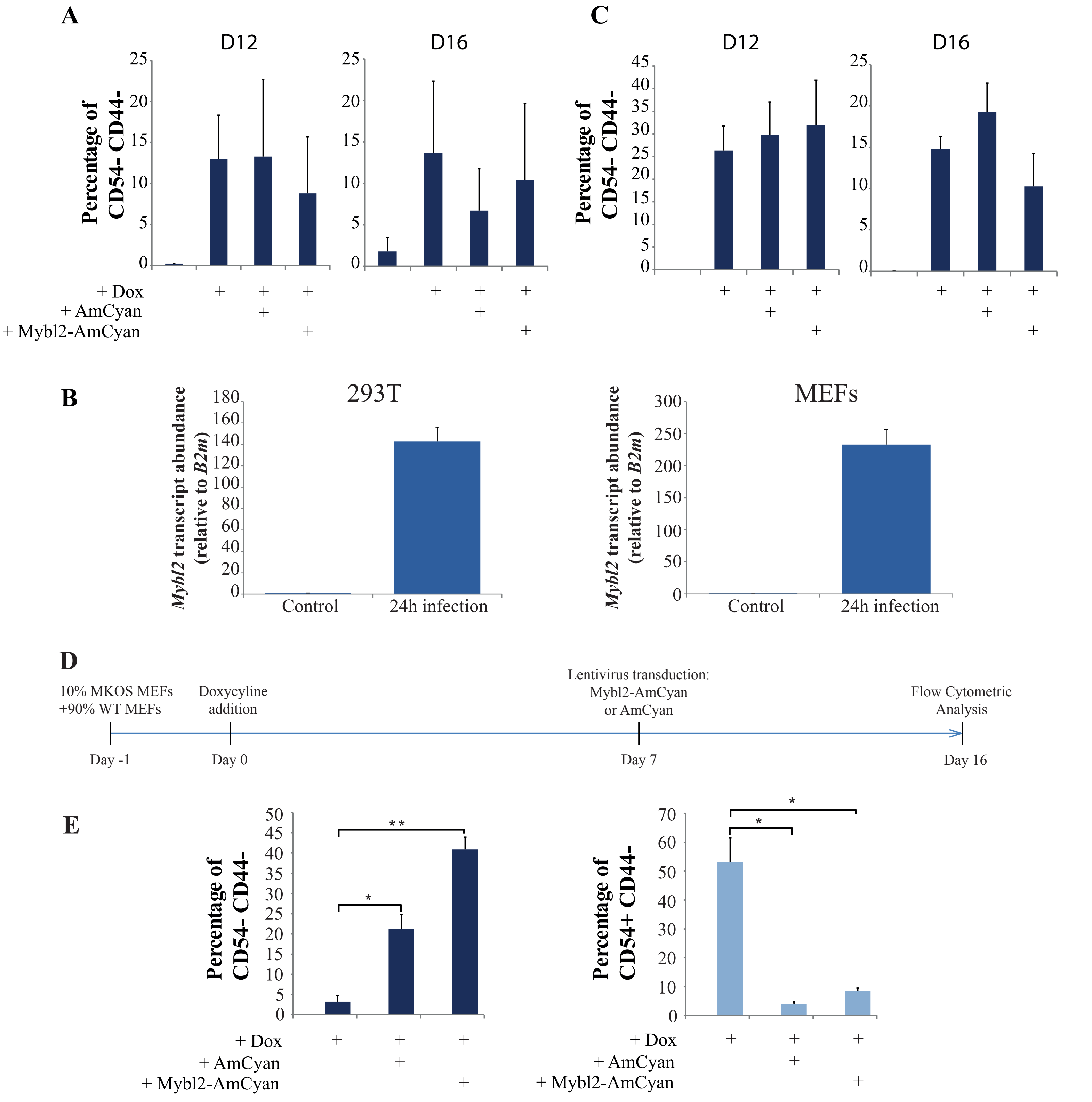

Figure S6. Mybl2 overexpression in Tg reprogrammable MEFs, Related to Figure 4. Graphs represent the percentange of $CD44^{-}$/$CD54^{-}$ cells (pre-iPSCs) at day 12 and 16 of reprogramming for Tg reprogrammable MEFs infected with AmCyan and Mybl2-AmCyan at day -2 before dox addition (A)or infected with AmCyan and Mybl2-Amcyan at day 3 after dox addition (C). N=3 biological replicates. (B) Validation of Mybl2 expression 24 hours after infection in 293T or MEFs (MOI 10). Mybl2 transcript abundance was measured by Taqman-PCR and normalized against the house keeping gene β2-microglobulin. N=1. (D) Schematic representation of the experimental design for Mybl2 infection at day 7 of somatic reprogramming in Tg reprogrammable MEFs. (E) Graphs showing the percentage of $CD44^{-}$/$CD54^{-}$ and $CD44^{-}$/$CD54^{+}$ in $mOrange^{+}$ (MKOS + dox) or in double positive $mOrange^{+}$, $AmCyan^{+}$ population (for MKOS + dox+ AmCyan and MKOS + dox + Mybl2AmCyan) at day 16. N=2 biological replicates.

Figure S7

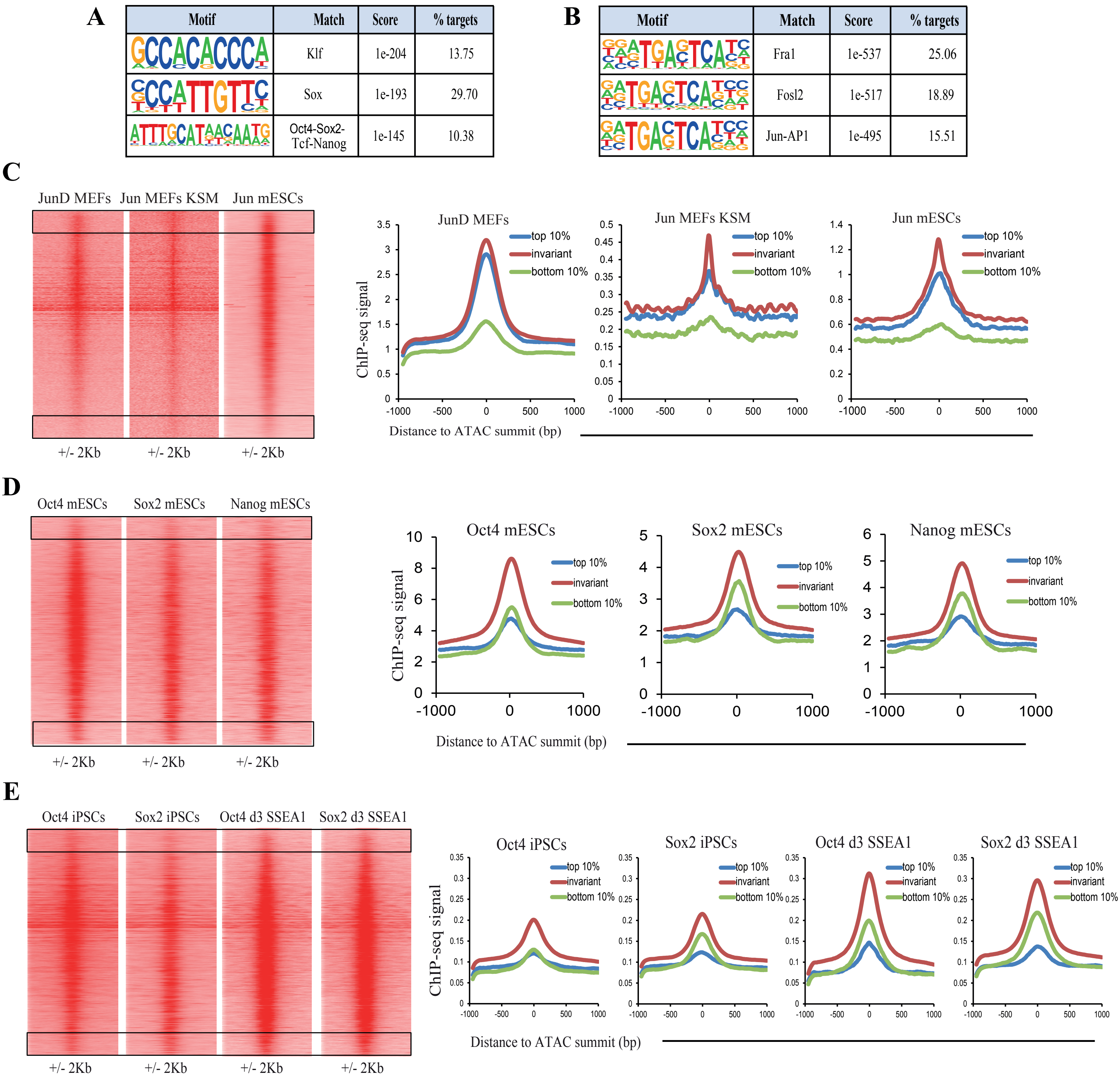

Figure S7. Mybl2 overexpression in Tg reprogrammable MEFs leads to a less accessible chromatin conformation, Related to Figure 5. (A) Motif enrichment in bottom 10% ATAC-seq peaks from union of peaks between control and Mybl2 overexpressing cells at day 3 of reprogramming.(B) Motif enrichment in top 10% ATAC-seq peaks from union of peaks between control and Mybl2 overexpressing cells at day 3 of reprogramming. (C) Heatmaps showing Jun ChIP-seq signal in (mESCs, MEFs, KSM+JunDN and MEFs) by decreasing ATAC-seq tag count signal for Mybl2-treated relative to control (AmCyan). (left) and average Jun ChIP-seq peak signal profiles with respect to the distance to ATAC summit (right). (D) Heatmaps showing Oct-4, Sox2 and Nanog ChIP-seq signal in mESCs, MEFs and iPSCs) by decreasing ATAC-seq tag count signal for Mybl2-treated relative to control (AmCyan) (right) and average Oct-4, Sox2 and Nanog ChIP-seq peak signal profiles with respect to the distance to ATAC summit (left). (E) Heatmaps showing Oct-4 and Sox2 ChIP-seq signal (in MEFs and iPSCs) by decreasing ATAC-seq tag count signal for Mybl2-treated relative to control (AmCyan) (right) and average Oct-4 and Sox2 ChIP-seq peak signal profiles with respect to the distance to ATAC summit (left).

Figure S8

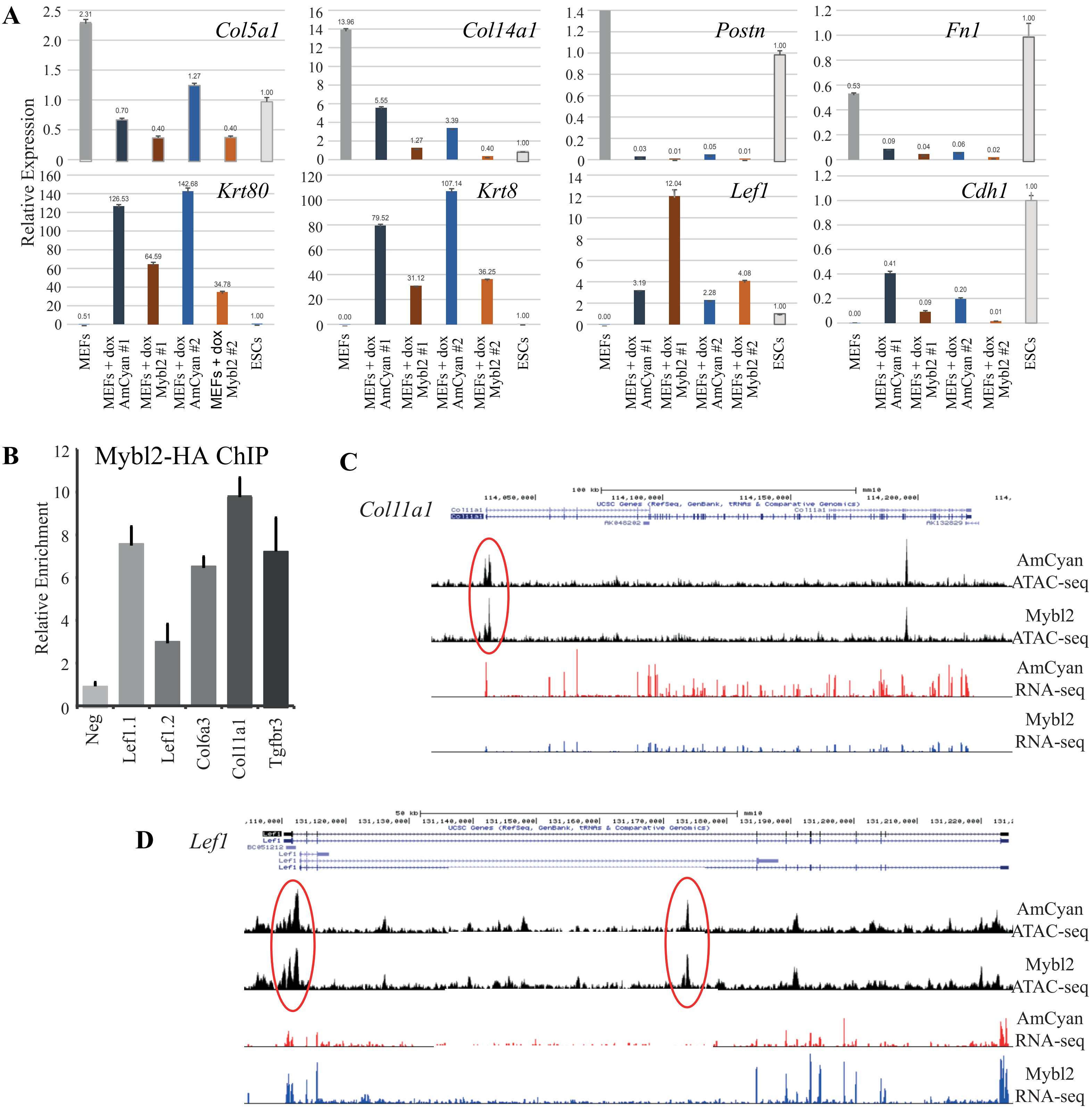

Figure S8. Mybl2 overexpression during reprogramming deregulates mesenchymal-to-epithelial transition (MET), Related to Figure 6. (A) Tg-reprogrammable MEFs were transduced with either AmCyan orMybl2-AmCyan lentivirus and induction of reprogramming was started one day after infection by doxycycline addition. Three days after the starting of reprogramming cells were sorted based on mOrange and AmCyan expression and RNA extracted. Taqman qPCR was done for the selected genes on two different RNA sets from the RNA-seq as well as for MEFs and ESCs. Results show relative gene expression normalized to the house keeping gene *β2-microglobulin.* 3 technical replicates and two biological replicates (set A and set B). ATAC-seq, and RNA-seq UCSC genome browser tracks for *Col11a1*(B) and *Lef1* C). Red circles show differential ATAC-seq peaks between both conditions. D) Representative X-ChIP on immortalized MEFs infected with Mybl2-HA-AmCyan using HA antibody. Enrichment fold was determined against negative control region. Graphs illustrate level of enrichment for each binding site identified for *Tgfbr3, Lef1, Col11a1, and Col6a2.*
